# DEPTOR suppresses lymphomagenesis by promoting EGFR degradation via HUWE1 E3 ligase

**DOI:** 10.1038/s41418-025-01497-5

**Published:** 2025-04-01

**Authors:** Xiufang Xiong, Xiaoyu Chen, Shengpeng Shao, Danrui Cui, Ruirui Qu, Baohui Wang, Ying Ma, Hui Pan, Yi Sun, Yongchao Zhao

**Affiliations:** 1 Department of Hepatobiliary and Pancreatic Surgery, the First Affiliated Hospital, Zhejiang University School of Medicine, Hangzhou, China; 2 Cancer Institute (Key Laboratory of Cancer Prevention and Intervention, China National Ministry of Education) of the Second Affiliated Hospital, Zhejiang University School of Medicine, Hangzhou, China; 3 Institute of Translational Medicine, Zhejiang University School of Medicine, Hangzhou, China; 4 Zhejiang Key Laboratory of Frontier Medical Research on Cancer Metabolism, Hangzhou, China; 5 Department of Medical Oncology, the First Affiliated Hospital, Zhejiang University School of Medicine, Hangzhou, China; 6 Zhejiang Provincial Key Laboratory of Pancreatic Disease, the First Affiliated Hospital, Zhejiang University School of Medicine, Hangzhou, China; 7 The First Affiliated Hospital of Zhejiang Chinese Medical University, Zhejiang Chinese Medical University, Hangzhou, China; 8 Department of Lung Transplantation, the First Affiliated Hospital, Zhejiang University School of Medicine, Hangzhou, China; 9 Department of Radiation Oncology, University of Michigan, Ann Arbor, MI USA

**Keywords:** Ubiquitylation, Tumour-suppressor proteins

## Abstract

DEPTOR, a naturally occurring inhibitor of mTOR, plays crucial roles in tumorigenesis and is frequently dysregulated in a variety of human cancers. Interestingly, DEPTOR could act either as a tumor suppressor or as an oncogene in a manner dependent of cellular context or tissue environment. Whether and how DEPTOR regulates lymphomagenesis remains elusive. In this study, we report that in a mouse lymphoma model induced by heterozygous *Pten* loss, *Deptor* knockout (KO) markedly accelerates lymphomagenesis, whereas degradation-resistant *Deptor*^*S275A*^ knock-in (KI) variant significantly inhibits it. Furthermore, *Deptor* KO mice spontaneously developed lymphomas in the later stages of their lifespan, and *Deptor* KO further shortened overall lifespan in *Pten*^*fl/fl*^*;MMTV-Cre* mice. Consistently, DEPTOR protein levels are significantly lower in human lymphoma tissues, as compared to normal lymph nodes. Mechanistically, DEPTOR, on one hand, enhances the interaction of EGFR to HUWE1 E3 ubiquitin ligase for targeted ubiquitination and proteasomal degradation, and subsequent inactivation of the MAPK signal. On the other hand, DEPTOR inactivates both mTORC1 and mTORC2 signals. Collectively, our study demonstrated that DEPTOR is a tumor suppressor that inhibits lymphomagenesis upon *Pten*-loss. The strategy that reactivates DEPTOR could be a promising approach for the treatment of lymphoma.

## Introduction

Lymphoma is a collection of malignant neoplasms that originate from lymphocytes, including B cells, T cells, and natural killer (NK) cells. These neoplasms have the potential to infiltrate lymphatic tissues, bone marrows, or extranodal sites. Comprising over 90 subtypes, lymphoma is conventionally divided into two main categories: Hodgkin lymphoma and non-Hodgkin lymphoma. Non-Hodgkin lymphoma encompasses a range of subtypes, including non-Hodgkin B-cell lymphomas, like Burkitt lymphoma and diffuse large B-cell lymphoma (DLBCL), as well as non-Hodgkin T-cell/NK-cell lymphomas [1]. The majority of lymphomas are classified as non-Hodgkin lymphomas, with nearly 95% of these cancers originating from B cells. B-cell receptor (BCR) signaling plays a pivotal role in maintaining the survival and promoting the proliferation of both normal B cells and most B-lymphoma cells. Currently, numerous clinical trials are underway to investigate agents that specifically target various components of BCR signaling pathway, as well as associated parallel pathways, for the treatment of diverse lymphoma subtypes. Prominent among these therapeutic strategies are inhibitors targeting specific isoforms of PI3K, mTOR, and Bruton’s Tyrosine kinase (BTK) [2–4].

The mTOR, a serine/threonine protein kinase, serves as a pivotal regulator of cell metabolism, growth, proliferation, survival, and autophagy, after integrating both intracellular and extracellular signals [5]. In mammalian cells, mTOR forms two complexes known as mTORC1 and mTORC2 [5, 6]. While mTORC1 activation promotes the synthesis of protein, lipid, nucleotides, and other macromolecules and inhibits autophagy by phosphorylating various effectors, including S6K1 and 4E-BP1, mTORC2 regulates cell survival, metabolism, cytoskeletal rearrangement and mobility through the direct phosphorylation and activation of AKT, SGK1, and PKC [5]. The activity of mTOR is tightly regulated by multiple signals, including growth factors, nutrients, energy, stress and hypoxia, via the PI3K/AKT pathway [5]. The PI3K/AKT/mTOR pathway is aberrantly activated in lymphomas, including in Hodgkin and non-Hodgkin lymphomas, as a result of anomalies in both the upstream and downstream regulators, such as mutation or gene amplification of the PI3K isoforms, PTEN loss, BCR signaling activation, AKT activation, overexpression of S6K, leading to proliferation, invasion and resistance [7]. Thus, PI3K/AKT/mTOR pathway is considered as an attractive target for effective lymphoma treatment [8].

DEPTOR is a naturally occurring mTOR inhibitor that directly suppresses both mTORC1 and mTORC2 [9, 10]. In cell culture settings, DEPTOR usually acts as a tumor suppressor; DEPTOR loss activates mTORC1 and mTORC2 and promotes growth and survival of many lines of cancer cells, including prostate [11] and lung [12] cancer cells. Under certain conditions (e.g., in some multiple myeloma), DEPTOR acts as an oncogene, since DEPTOR inhibition of mTORC1 relieves the feedback inhibition from S6K1 to PI3K, boosting AKT activity for cancer cells survival [9, 10]. However, the in vivo role of DEPTOR in lymphomagenesis is yet to be well understood.

In this study, we generated a *Deptor*^*S275A*^ mutant knock-in (KI) mouse that expresses a more stable form of DEPTOR, theoretically exerting greater inhibition on mTOR activity. Using this *Deptor* KI mouse model in combination with heterozygous *Pten* deletion, we found that DEPTOR KI significantly inhibited lymphomagenesis induced by *Pten* heterozygous loss. Conversely, *Deptor* knockout (KO) markedly promoted lymphomagenesis driven by *Pten* heterozygous loss and shortened the overall lifespan in *Pten*^*fl/fl*^*;MMTV-Cre* mice. Additionally, DEPTOR protein levels were reduced in human lymphoma tissues. Mechanistically, DEPTOR binds to HUWE1, an E3 ubiquitin ligase for EGFR, facilitating the interaction between HUWE1 and EGFR, leading to the proteasomal degradation of EGFR and subsequent inactivation of the ERK1/2 pathway, thereby inhibiting lymphomagenesis. Thus, our study provides strong evidence that DEPTOR functions as a tumor suppressor in lymphomagenesis upon *Pten* loss.

## Results

### DEPTOR^S275A^ suppresses lymphomagenesis triggered by *Pten* heterozygous loss in vivo

We and the others previously found that DEPTOR is a substrate of SCF^β-TrCP^ E3 ubiquitin ligase [13–15]. Upon growth factor exposure, DEPTOR was phosphorylated at putative binding site for β-TrCP (D-pS-G-X-X-pS) on codons 286–291 by RSK1/S6K1 kinases and then recognized by β-TrCP for targeted degradation by SCF E3 ligase, leading to mTOR activation [13]. Interestingly, DEPTOR acts either as an oncogene or as a tumor suppressor in a cellular context dependent manner [10, 16]. To elucidate in vivo function of DEPTOR (e.g. acting as a tumor suppressor vs. an oncogene) and its potential regulation of tumorigenesis via modulating PI3K/mTOR/AKT pathway, we generated a knock-in (KI) mouse model expressing DEPTOR^S275A^ (equivalent to human S287A) (Fig. S1A), a DEPTOR mutant resistant to SCF^β-TrCP^-mediated degradation [13]. We confirmed the genotype of the mice by PCR genotyping (Fig. S1B) and the S275A mutation by sequencing the tail DNA from 6 *Deptor*^*S275A/S275A*^ mice (Fig. S1C). Immunoblotting (IB) also confirmed the accumulation of DEPTOR protein in the primary MEFs and brains of *Deptor*^*S275A/S275A*^ mice (Fig. S1D, E). We observed a moderate elevation in DEPTOR protein levels in the primary MEFs and brains of *Deptor*^*S275A/+*^ mice and a significant increase in *Deptor*^*S275A/S275A*^ mice (Fig. S1D, E). However, DEPTOR mRNA levels were only significantly elevated in the primary MEFs and brains of *Deptor*^*S275A/S275A*^ mice (Fig. S1D, E). The fold increase in DEPTOR protein was greater than that of mRNA, suggesting that the elevated protein levels may result from both enhanced transcription and reduced protein degradation. As to how DEPTOR transcription is elevated in *Deptor*^*S275A/S275A*^ mice, we hypothesize that DEPTOR stabilization inhibits mTOR, which typically represses DEPTOR transcription [9]. Finally, DEPTOR^S275A/S275A^ protein exhibits a significant prolongation of its half-life (Fig. S1F). Collectively, these results demonstrate the successful generation of *Deptor* KI mouse model. The genotyping of 188 offspring at age of 5 weeks or older from the intercrossing of *Deptor*^*S275A/+*^ mice revealed that the ratio of *Deptor*^*+/+*^: *Deptor*^*S275A/+*^: *Deptor*^*S275A/S275A*^ was approximately 1: 2: 1 (Fig. S1G), indicating that homozygous *Deptor*^*S275A*^ KI mice are viable. Moreover, the body weight of the female and male mice at the age of 3 weeks showed no significant difference among the three genotypes (Fig. S1H). These results indicate that DEPTOR^S275A^ mutant has no effects on embryonic development and postnatal growth in mice.

**Fig. 1:**
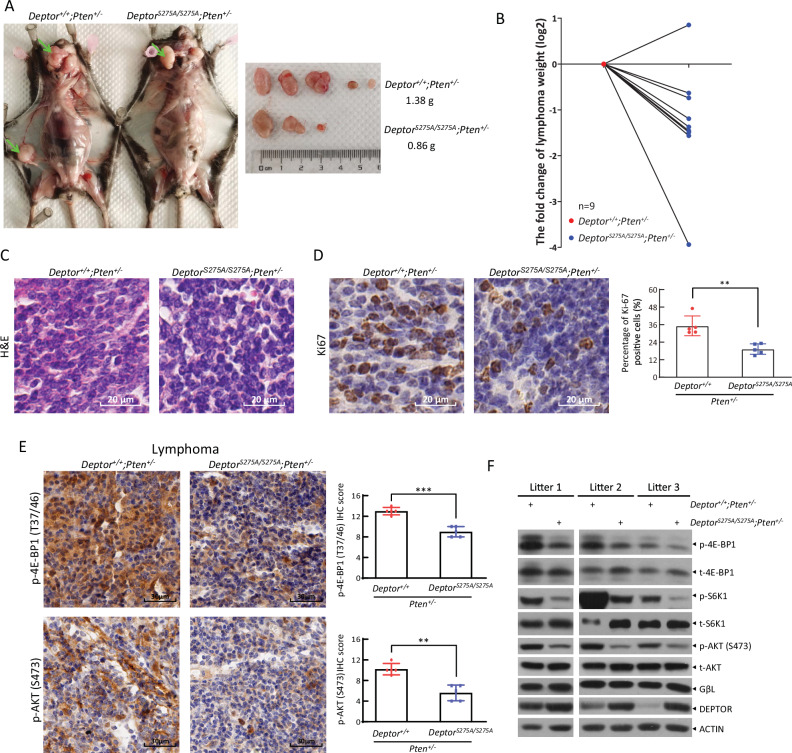
DEPTOR^S275A^ suppresses lymphomagenesis triggered by *Pten* heterozygous loss in vivo. **A**‒**D** DEPTOR^S275A^ suppresses lymphomagenesis triggered by *Pten* heterozygous loss. Whole body necropsy of a representative *Deptor*^*+/+*^*;Pten*^*+/−*^ and *Deptor*^*S275A/S275A*^*;Pten*^*+/−*^ littermate female mouse at the age of 8 months (left, **A**). The image and weight of the lymphomas from the mice (left, **A**) are shown (right, **A**). The weight of lymphomas from *Deptor*^*S275A/S275A*^*;Pten*^*+/−*^ mice is expressed as the Log2-fold change compared with that from *Deptor*^*+/+*^*;Pten*^*+/−*^ littermate mice (**B**). *n* = 9 for each group. The lymphomas from littermate mice with indicated genotypes were sectioned and then subjected to H&E staining (**C**) and immunohistochemical (IHC) staining with Ki67 antibody (**D**). Scale bar: 20 µm. **E**, **F** DEPTOR^S275A^ inactivates mTORC1 and mTORC2 signaling in *Pten*^*+/−*^ mice. The lymphomas of paired mice from different litters with indicated genotypes were subjected to IHC staining (**E**) or immunoblotting (IB) (**F**) with the indicated antibodies (Abs). Scale bar: 30 µm. The staining quantification was determined by IHC scoring using an IRS system from five random fields of lymophoma sections (right, **D** & **E**). **p < 0.01; ***p < 0.001.

The viability of *Deptor*^*S275A/S275A*^ mice gave us an opportunity to study whether DEPTOR accumulation would cause a constitutive inactivation of mTOR, thus blocking tumorigenesis induced by *Pten* loss, which activates the PI3K/AKT/mTOR signal. Heterozygous deletion of *Pten* in mice is known to induce lymphomagenesis with high frequency and cause sporadic tumors in multiple tissues, including endometrium, breast, prostate, gastrointestinal tract, thyroid, and liver [17–19]. We then crossed *Deptor*^*S275A/S275A*^ mice with *Pten*^*+/−*^ mice to generate *Deptor*^*S275A/S275A*^*;Pten*^*+/−*^ and *Deptor*^*+/+*^*;Pten*^*+/−*^ mice for monitoring tumor development triggered by *Pten* heterozygous loss. Consistently, we observed that mice with both of genotypes had enlarged lymph nodes at the cervical and inguinal sites at the age of 8 months (Fig. 1A). Importantly, the size and weight of lymph nodes from *Deptor*^*S275A/S275A*^*;Pten*^*+/−*^ mice were reduced, compared with those from *Deptor*^*+/+*^*;Pten*^*+/−*^ littermates (Fig. 1A, B). The H&E staining of enlarged lymph nodes from *Deptor*^*+/+*^*;Pten*^*+/−*^ mice exhibited disorganized cells with nuclei of varying sizes, some having darker staining, indicative of pathological changes including atypia and neoplastic lesions (Fig. 1C). Conversely, lymphomas from *Deptor*^*S275A/S275A*^*;Pten*^*+/−*^ mice revealed relatively orderly arranged cells and nuclei with relatively uniformed size and regular shape (Fig. 1C) with reduced Ki67 staining (Fig. 1D), suggesting that KI of *Deptor*^*S275A*^ significantly suppresses lymphomagenesis triggered by *Pten* heterozygous loss. Addtionally, immunohistochemistry (IHC) staining revealed that the lymphomas arising in *Deptor*^*+/+*^*;Pten*^*+/−*^ and *Deptor*^*S275A/S275A*^*;Pten*^*+/−*^ mice include both T-cell and B-cell lymphomas, as evidenced by positive staining of CD3, a T-cell marker, and B220, a B-cell marker, respectively (Fig. S2A). This is consistent with previous studies demonstrating that *Pten*^*+/−*^ mice develop both T-cell and B-cell lymphomas [17, 20].

Our previous study showed that *Deptor* knockout promotes prostate tumorigenesis triggered by *Pten* loss via the activation of mTORC1/2 signals [11]. Thus, we next determined whether the mTORC1/2 signals are inactivated by KI of this degradation-resistant DEPTOR mutant in lymphomagenesis. Indeed, the IHC staining showed that phosphorylation of 4E-BP1 and AKT, the downstream effectors of the mTORC1 and mTORC2 signals, respectively, were significantly reduced in the lymphomas from *Deptor*^*S275A/S275A*^*;Pten*^*+/−*^ mice, as compared to those from *Deptor*^*+/+*^*;Pten*^*+/−*^ littermates (Fig. 1E). Consistently, the IB analysis confirmed higher level of DEPTOR protein, but lower levels of phosphorylated 4E-BP1, S6K1, and AKT in the lymphomas from *Deptor*^*S275A/S275A*^*;Pten*^*+/−*^ mice (Fig. 1F). Thus, the degradation-resistant DEPTOR^S275A^ mutant suppresses lymphomagenesis via the inactivation of mTORC1/2 signals in *Pten*^*+/−*^ mice.

### DEPTOR^S275A^ reduces EGFR levels and inactivates the RAS/MAPK signaling

**Fig. 2:**
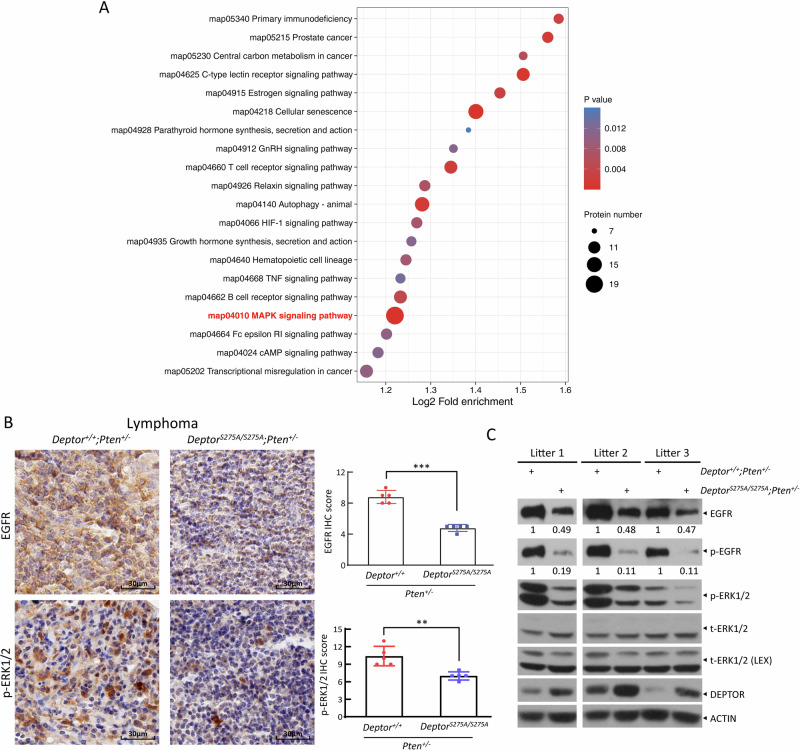
DEPTOR^S275A^ reduces EGFR levels and inactivates the RAS/MAPK signaling. **A** MAPK signaling pathway is downregulated in lymphomas from *Deptor*^*S275A/S275A*^*;Pten*^*+/−*^ mice. The lymphomas from *Deptor*^*+/+*^*;Pten*^*+/−*^ and *Deptor*^*S275A/S275A*^*;Pten*^*+/−*^ littermate mice were subjected to label-free quantitative mass spectrometry. The top 20 downregulated signaling pathways, identified through enrichment analysis of differentially expressed proteins, are presented. The color of the dots indicates the significance of enrichment (*p*-value), with red representing higher enrichment significance, and the size of the dots represents the number of differentially expressed proteins in the pathway. **B**, **C** DEPTOR^S275A^ reduces EGFR levels and inactivates the RAS/MAPK signal. The lymphomas of paired mice from different litters with indicated genotypes were subjected to IHC staining (**B**) or IB (**C**) with the indicated Abs. Representative images of IHC staining are shown (left, **B**). Scale bar: 30 µm. The staining quantification was determined by IHC scoring using an IRS system from five random fields of lymophoma sections (right, **B**). **p < 0.01; ***p < 0.001. The band density was quantified using ImageJ and expressed as fold change relative to the control, with the control value arbitrarily set to 1.

To explore whether other mechanisms are involved in the suppression of lymphomagenesis by DEPTOR^S275A^ mutant, we harvested the lymphomas from littermate mice and performed label-free quantitative mass spectrometry to identify the altered signaling pathways. The enrichment analysis of differentially expressed proteins revealed significant downregulation in the MAPK signaling pathway, which has the highest number of differentially expressed proteins among all significantly downregulated pathways in lymphomas from *Deptor*^*S275A/S275A*^*;Pten*^*+/−*^ mice (Fig. 2A). We further confirmed the inactivation of the RAS/MAPK signaling in *Deptor*^*S275A/S275A*^*;Pten*^*+/−*^ lymphomas by IHC staining (Fig. 2B) and IB analysis (Fig. 2C), as evidenced by reduced staining and levels of phosphorylated ERK1/2. Notably, EGFR, an upstream receptor tyrosine kinase (RTK) that activates both the RAS/MAPK and PI3K/AKT/mTOR pathways, was among the differentially expressed proteins, with its levels significantly reduced in the lymphomas from *Deptor*^*S275A/S275A*^*;Pten*^*+/−*^ mice (Table S1). Furthermore, we confirmed that EGFR levels were significantly reduced in the lymphomas from *Deptor*^*S275A/S275A*^*;Pten*^*+/−*^ mice by both IHC staining and IB analysis (Fig. 2B, C). In addition, EGFR levels were also reduced in the primary MEFs from *Deptor*^*S275A/S275A*^*;Pten*^*+/−*^ mice (Fig. S3A). Thus, the expression of the degradation-resistant DEPTOR^S275A^ mutant appears to down-regulate EGFR levels and to attenuate MAPK signaling.

### DEPTOR^S275A^ promotes EGFR degradation via proteasome

**Fig. 3:**
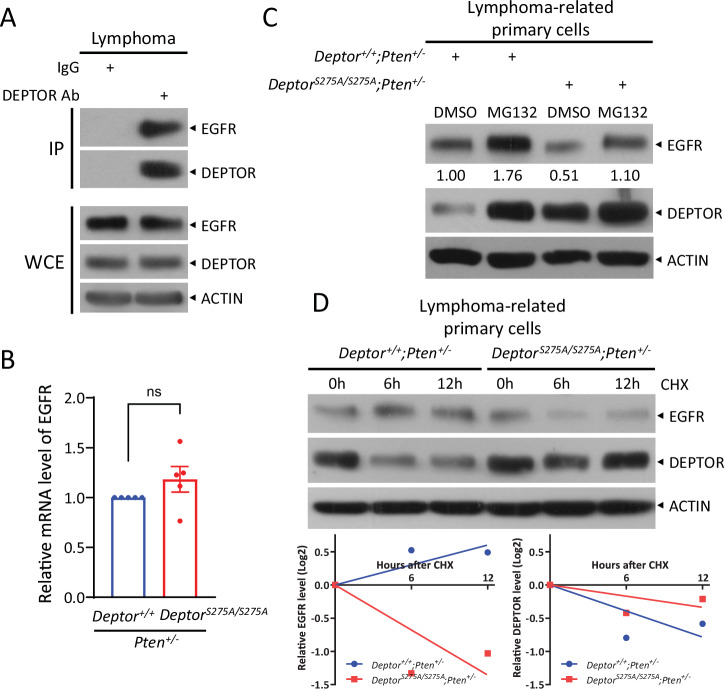
DEPTOR^S275A^ promotes EGFR degradation via proteasome. **A** DEPTOR binds to EGFR in lymphomas. The lymphomas from *Deptor*^*+/+*^*;Pten*^*+/−*^ mice were lysed and subjected to immunoprecipitation (IP) with anti-DEPTOR Ab, followed by IB with indicated Abs. WCE: whole cell extracts. **B** DEPTOR^S275A^ has no effect on EGFR mRNA levels. The lymphomas from five pairs of mice with indicated genotypes were harvested for qRT-PCR analysis. mean ± S.E.M; *n* = 5; ns: not significant. **C** MG132 blocks EGFR reduction in *Deptor*^*S275A/S275A*^*;Pten*^*+/−*^ lymphoma-related primary cells. Cells derived from lymphomas from two pairs of mice with indicated genotypes were treated with MG132 for 6 h before being harvested for IB analysis. The band density was quantified by ImageJ and expressed as the relative gray value (compared with cells from *Deptor*^*+/+*^*;Pten*^*+/−*^ treated with DMSO) below the band. **D** DEPTOR^S275A^ shortens the protein half-life of EGFR. Cells derived from lymphomas from paired mice with indicated genotypes were treated with CHX for various time periods and then harvested for IB analysis (top). The band density was quantified by ImageJ, and the decay curves are shown (bottom).

In our previous study, DEPTOR bind to the kinase domain of EGFR through its PDZ domain, thereby inactivating the EGFR signaling without affecting EGFR levels in lung cancer cells [12]. To explore the underlying mechanism by which DEPTOR reduces EGFR levels in lymphomas, we first determined if DEPTOR binds to EGFR and found that EGFR was readily detected in the DEPTOR immunoprecipitates from the lysate of lymphomas (Fig. 3A). Next, DEPTOR KI did not affect the mRNA levels of EGFR in the lymphomas (Fig. 3B). Furthermore, we harvested the lymphoma tissues from two pairs of littermates with genotypes of *Deptor*^*+/+*^*;Pten*^*+/−*^ and *Deptor*^*S275A/S275A*^*;Pten*^*+/−*^, and trypsinized them to obtain lymphoma-related primary cells. In *Deptor*^*S275A/S275A*^*;Pten*^*+/−*^ lymphoma-related primary cells, the greater reduction of EGFR protein levels were significantly blocked by proteasome inhibitor MG132 (Fig. 3C), and the half-life of EGFR protein was much shortened (Fig. 3D). Since EGFR activation requires its interaction with ligands at the cell surface, we isolated cell membranes from primary MEFs and observed that EGFR levels on the membranes of *Deptor*^*S275A/S275A*^*;Pten*^*+/−*^ MEFs were significantly reduced. However, treatment with MG132 effectively restored these diminished EGFR levels (Fig. S3B). Together, our results indicate that DEPTOR^S275A^ promotes EGFR degradation, likely through proteasome.

### DEPTOR facilitates the interaction between HUWE1 and EGFR

**Fig. 4:**
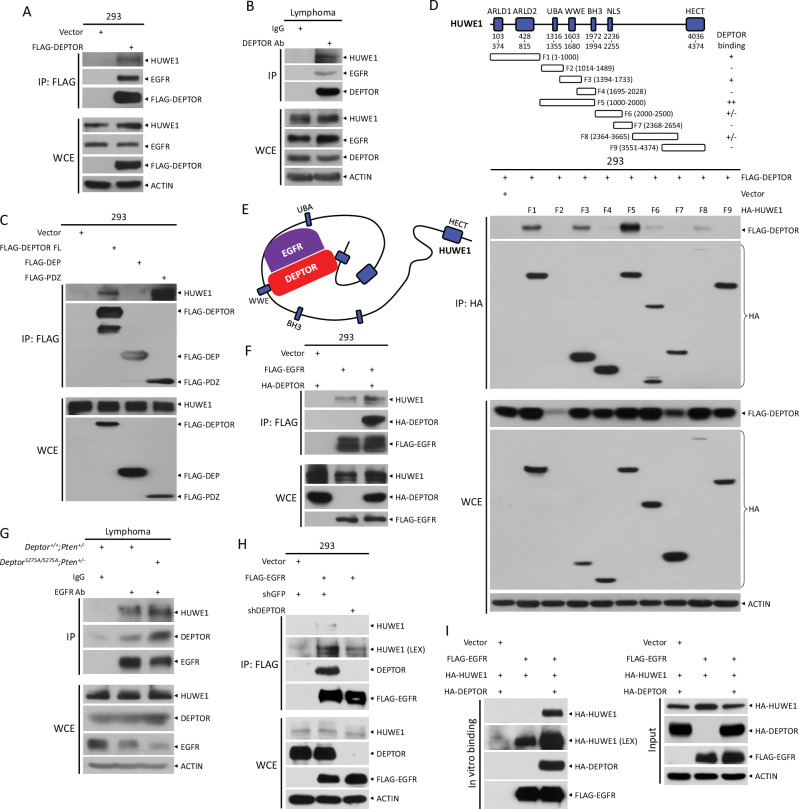
DEPTOR facilitates the interaction between HUWE1 and EGFR. **A** Ectopically expressed DEPTOR binds to HUWE1 and EGFR. HEK293 cells were transfected with indicated plasmids for 48 h and then subjected to IP with FLAG beads, followed by IB with indicated Abs. **B** DEPTOR binds to HUWE1 and EGFR in lymphomas. The lymphomas from *Deptor*^*+/+*^*;Pten*^*+/−*^ mice were lysed and subjected to IP with anti-DEPTOR Ab, along with normal IgG as a control, followed by IB with indicated Abs. **C** DEPTOR binds to HUWE1 via its PDZ domain. HEK293 cells were transfected with indicated plasmids for 48 h and then subjected to IP with FLAG beads, followed by IB with indicated Abs. **D** DEPTOR binds to the N-terminal portion of HUWE1. HEK293 cells were transfected with indicated plasmids for 48 h and then subjected to IP with anti-HA Ab, followed by IB with indicated Abs. **E** A model for DEPTOR binding with HUWE1 and EGFR. **F** DEPTOR overexpression promotes EGFR binding to HUWE1. HEK293 cells were transfected with indicated plasmids for 48 h and then subjected to IP with FLAG beads, followed by IB with indicated Abs. **G** DEPTOR^S275A^ promotes EGFR binding to HUWE1. The lymphomas from paired mice were lysed and subjected to IP with anti-EGFR Ab, followed by IB with indicated Abs. **H** DEPTOR knockdown inhibits EGFR binding to HUWE1. HEK293 cells infected with indicated lentivirus were transfected with indicated plasmids for 48 h and then subjected to IP with FLAG beads, followed by IB with indicated Abs. **I** In vitro binding assay shows that DEPTOR facilitates the interaction between HUWE1 and EGFR. The details of the in vitro binding assay were described in the Material and Methods section. LEX longer exposure, WCE whole cell extracts.

To elucidate the mechanism by which DEPTOR^S275A^ mutant enhances EGFR degradation, we deployed an affinity purification coupled with mass spectrometry approach to identify the binding protein of DEPTOR that is involved in protein degradation. Interestingly, in the FLAG-DEPTOR immunoprecipitates, we identified HUWE1 (Fig. S4), an E3 ubiquitin ligase, previously shown to protect mice from kidney injury by targeting EGFR for ubiquitination and degradation [21]. Indeed, FLAG-DEPTOR bound to HUWE1 in HEK293 cells (Fig. 4A), and more importantly, the DEPTOR-HUWE1-EGFR forms a complex, since both HUWE1 and EGFR were pulled down by anti-DEPTOR antibody from the lymphoma lysates (Fig. 4B). We next defined the binding domains between DEPTOR and HUWE1 by the domain truncation strategy. Our previous study showed that DEPTOR binds to EGFR via its PDZ domain at the C-terminus [12]. Interestingly, the same DEPTOR domain also mediated the HUWE1 binding (Fig. 4C). HUWE1, a member of the largest human HECT E3 ligase family, comprises a C-terminal catalytic HECT domain and an N-terminal giant ring-like scaffold crucial for substrate binding [22]. We next mapped the interaction domain in HUWE1 for DEPTOR binding by ectopically expressing a serial of truncate mutants of HUWE1 [23] in HEK293 cells (Fig. 4D), and found that several fragments at the N-terminal region of HUWE1, specifically F1 (1‒1000), F3 (1394‒1733), and F5 (1000‒2000), were involved in DEPTOR binding (Fig. 4D). Given that the N terminal ring-like structure of HUWE1 encompasses diverse protein-protein interaction modules, our result suggests that DEPTOR binding likely facilitates the association of HUWE1 to EGFR (Fig. 4E). Indeed, co-transfection of a construct expressing HA-DEPTOR enhanced the binding of FLAG-EGFR with endogenous HUWE1 (Fig. 4F). Furthermore, in the lymphomas from *Deptor*^*S275A/S275A*^*;Pten*^*+/−*^ mice, EGFR antibody was able to pull down a greater amount of HUWE1 compared to those from *Deptor*^*+/+*^*;Pten*^*+/−*^ lymphomas (Fig. 4G), indicating an enhanced binding of HUWE1 with EGFR by DEPTOR^S275A^. Conversely, lentiviral expression of shDEPTOR, which almost completely abolished DEPTOR protein levels, significantly reduced the binding between FLAG-EGFR and HUWE1, but not completely eliminated it (Fig. 4H). The in vitro binding assay further revealed that, in the absence of exogenous HA-DEPTOR, FLAG-EGFR still bound to HA-HUWE1. However, the presence of HA-DEPTOR significantly enhanced this binding (Fig. 4I). Collectively, these results suggest that the binding of DEPTOR to the N-terminal region of HUWE1, via its PDZ domain, facilitates the interaction between HUWE1 and EGFR.

### DEPTOR protein levels are reduced in human lymphoma tissues

**Fig. 5:**
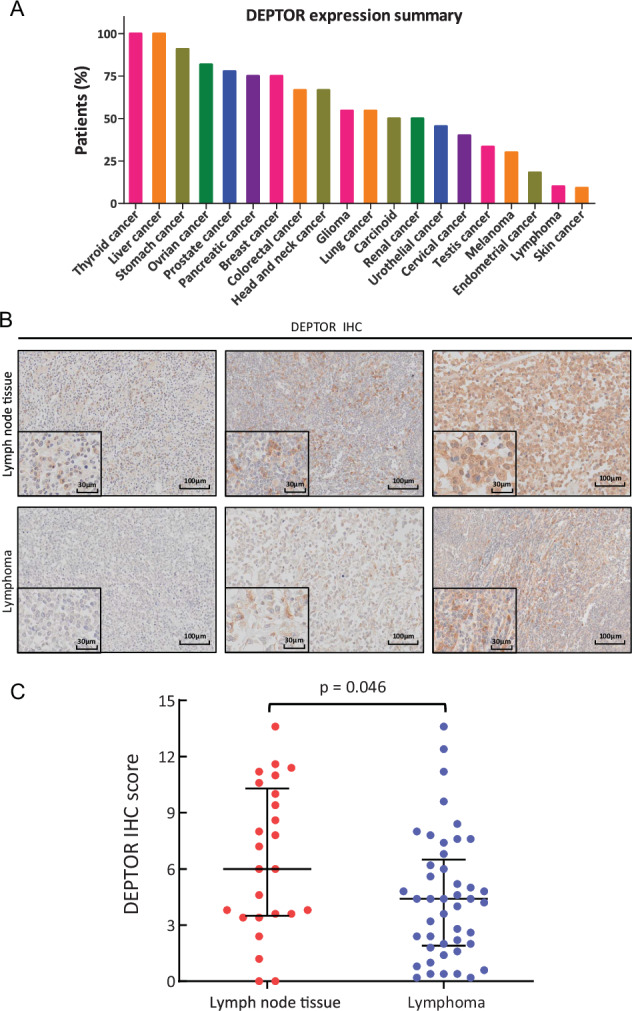
DEPTOR protein levels are reduced in human lymphoma tissues. **A** DEPTOR level is low in lymphoma. The Human Protein Atlas data (https://www.proteinatlas.org) are used to show the percentages of cases with high or medium DEPTOR level by IHC staining in various human cancers. **B**, **C** DEPTOR protein levels in human lymphoma tissues are lower than in normal lymph node tissues. Lymphoma tissue microarray, consisting of 45 lymphoma tissues and 25 normal lymph node tissues, was immunostained for DEPTOR levels. Shown are representative images of DEPTOR staining (**B**). Scale bars: 100 μm or 30 μm (inset). The staining quantification was determined by IHC scoring using an IRS system according to the staining intensity and the percentage of positive cells (**C**). *p* = 0.046, Wilcoxon rank sum test.

To determine the association of DEPTOR with human lymphomagenesis, we searched the Human Protein Atlas data (https://www.proteinatlas.org), and found that the lymphoma tissues with positive DEPTOR staining had the lowest percentage among the patients with many other malignancies, including thyroid cancer, liver cancer, stomach cancer, ovarian cancer and so on (Fig. 5A). Specifically, only 10% of patients with lymphoma exhibited medium expression of DEPTOR protein, while the remaining 90% of cases were negative for staining in their bone marrow and lymphoid tissues, suggesting that DEPTOR expression is likely to be decreased during human lymphomagenesis. Consistently, DEPTOR expression in lymphoid diffuse large B-cell lymphoma (DLBC) is nearly the lowest across 33 TCGA tumors (Fig. S5). To further confirm the reduction of DEPTOR levels in human lymphomas, we performed the IHC staining of human lymphoma tissue microarrays using an anti-DEPTOR antibody with good specificity [11] (Fig. 5B). The lymphoma tissue microarrays consist of 45 lymphoma tissues, including 40 cases of diffuse large B-cell lymphoma and 5 cases of follicular lymphoma, and 25 normal lymph node tissues. The IHC scores based on the staining intensity and the percentage of cells with positive DEPTOR staining showed that DEPTOR levels in lymphoma tissues were significantly lower, compared to those in normal lymph node tissues (Fig. 5C), by statistical analysis with Wilcoxon rank sum test. Thus, the levels of DEPTOR protein are downregulated in human lymphoma tissues.

### DEPTOR disruption promotes lymphomagenesis in vivo

**Fig. 6:**
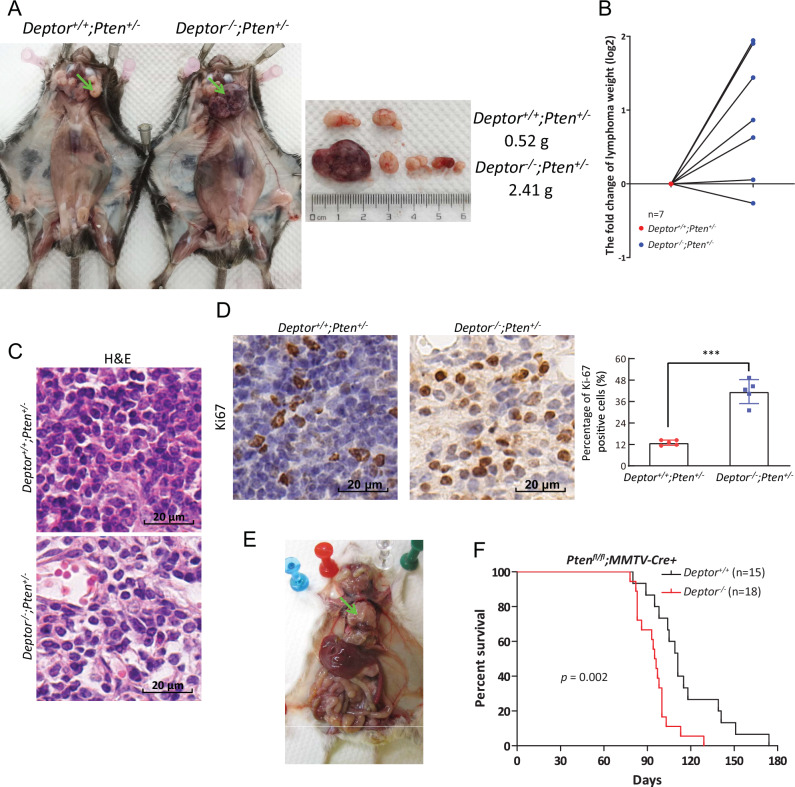
DEPTOR disruption promotes lymphomagenesis in vivo. **A**, **D** DEPTOR disruption promotes the formation of lymphoma triggered by *Pten* heterozygous loss. Whole body necropsy of a representative *Deptor*^*+/+*^*;Pten*^*+/−*^ and *Deptor*^*−/−*^*;Pten*^*+/−*^ littermate male mouse at the age of 9 months (left, **A**). The image and weight of the lymphomas from the mice (left, **A**) are shown (right, **A**). The weight of lymphomas from *Deptor*^*−/−*^*;Pten*^*+/−*^ mice is expressed as the Log2-fold change compared with that from *Deptor*^*+/+*^*;Pten*^*+/−*^ littermate mice (**B**). n = 7 for each group. The lymphomas from littermate mice with indicated genotypes were sectioned and then subjected to H&E staining (**C**) and IHC staining with Ki67 Ab. Scale bar: 20 µm. The staining quantification was determined by IHC scoring using an IRS system from five random fields of lymophoma sections (right, **D**). ***p < 0.001. **E**, **F** DEPTOR disruption promotes thymic lymophoma formation and shortens the life-span in *Pten*^*fl/fl*^*;MMTV-Cre* mice. Whole body necropsy of a representative *Deptor*^*−/−*^*;Pten*^*fl/fl*^*;MMTV-Cre* male mouse at the age of 3 months is shown (**E**). An arrow points to the enlarged thymus. Kaplan–Meier survival curves of *Pten*^*fl/fl*^*;MMTV-Cre* male mice with indicated *Deptor* genotypes (**F**). *p* = 0.002, log-rank test.

Given the downregulation of DEPTOR in human lymphoma tissues, we further determined if DEPTOR disruption would have any effect on lymphomagenesis under *Pten*^*+/−*^ background in vivo using a *Deptor* knockout mouse model [11]. We obtained paired *Deptor*^*+/+*^*;Pten*^*+/−*^ and *Deptor*^*−/−*^*;Pten*^*+/−*^ littermates and observed that both the size and weight of lymphomas from *Deptor*^*−/−*^*;Pten*^*+/−*^ mice were increased, compared with those from *Deptor*^*+/+*^*;Pten*^*+/−*^ littermates (Fig. 6A, B). Moreover, H&E staining revealed lymphomas from *Deptor*^*−/−*^*;Pten*^*+/−*^ mice have a high degree of malignancy, characterized by a greater variability in cell size and shape as well as a more chaotic cell arrangement, in contrast to the relatively uniform cell size and shape, and the closely packed yet orderly cell arrangement observed in lymphomas from *Deptor*^*+/+*^*;Pten*^*+/−*^ littermates (Fig. 6C). Consistently, more proliferating cells with Ki67 positive staining were observed in lymphomas from *Deptor*^*−/−*^*;Pten*^*+/−*^ mice (Fig. 6D). Thus, DEPTOR disruption significantly promotes lymphomagenesis triggered by *Pten* heterozygous loss. Additionally, similar to the *Deptor*^*+/+*^*;Pten*^*+/−*^ mice, the lymphomas that develop in *Deptor*^*−/−*^*;Pten*^*+/−*^ mice comprise both T-cell and B-cell lymphomas (Fig. S2B).

In addition, we monitored a total of 5 *Deptor*^*+/+*^ and 12 *Deptor*^*−/−*^ female mice for lymphomagenesis for a period of 24 months. Whole body necropsy found that 20% (1/5) of *Deptor*^*+/+*^ and 66.7% (8/12) of *Deptor*^*−/−*^ female mice spontaneously developed lymphomas, with enlarged lymph nodes in neck, mesentery and thymus (Fig. S6A). Histologic analysis and IHC staining confirmed the diagnosis of T-cell and B-cell lymphomas, as evidenced by positive staining of CD3 and B220, respectively (Fig. S6B). High levels of Cre recombinase have been found in B cells and T cells of *MMTV-Cre* mice (www.jax.org/strain/003553), and *Pten*^*fl/fl*^*;MMTV-Cre* mice succumbed by 14 weeks due to the development of thymic lymphomas [24]. We then crossed *Deptor*^*-/-*^ mice with *Pten*^*fl/fl*^*;MMTV-Cre* mice to generate paired compound mice with the genotypes of experimental *Deptor*^*-/-*^*;Pten*^*fl/fl*^*;MMTV-Cre* and control *Deptor*^*+/+*^*;Pten*^*fl/fl*^*;MMTV-Cre*. Indeed, all of these mice developed thymic lymphomas with enlarged thymus, and perished by the age of 6 months (Fig. 6E, F). Notably, DEPTOR disruption significantly shortened the life span of *Pten*^*fl/fl*^*;MMTV-Cre* mice (Fig. 6F). Collectively, DEPTOR disruption promotes lymphomagenesis in mice.

### DEPTOR disruption induces EGFR levels and activates MAPK and mTORC1/2 pathways during lymphomagenesis

**Fig. 7:**
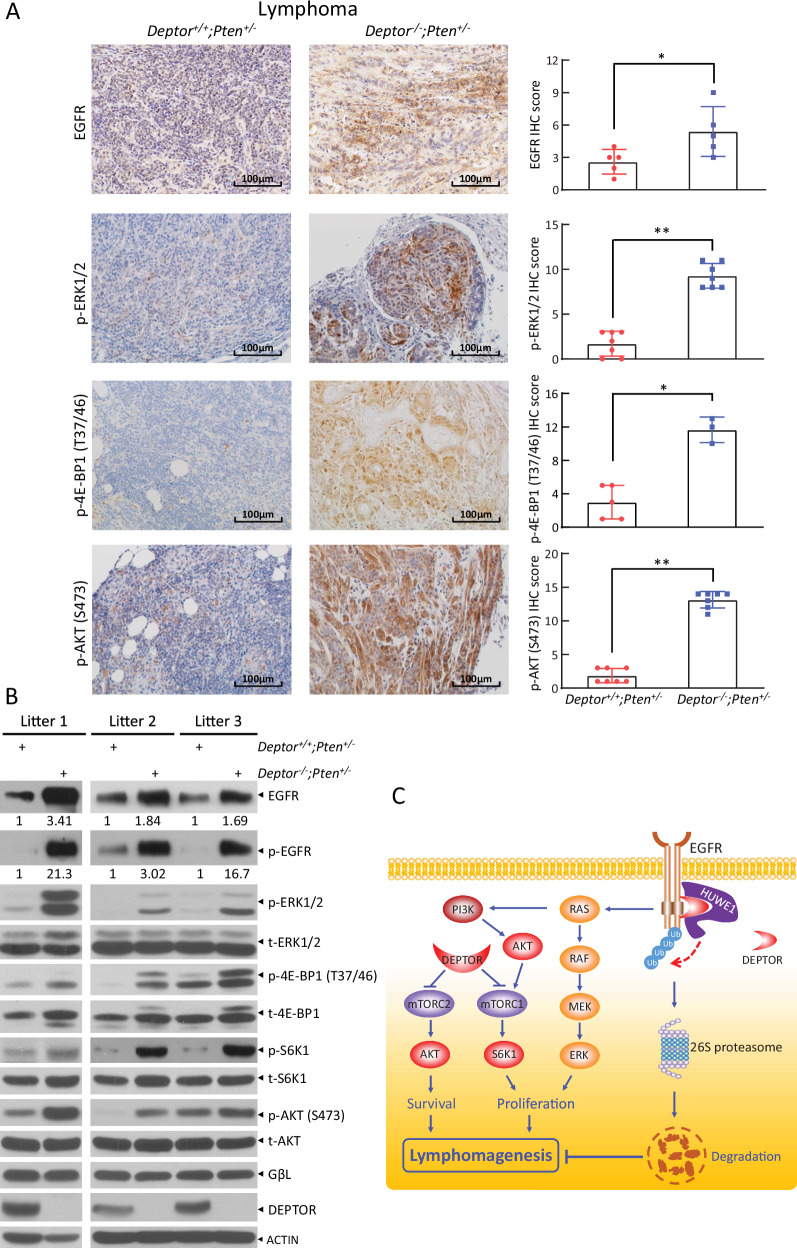
DEPTOR disruption induces EGFR levels and activates MAPK and mTORC1/2 pathways in lymphomagenesis. **A**, **B** DEPTOR disruption induces EGFR levels and activates MAPK and mTORC1/2 pathways. The lymphomas from *Deptor*^*+/+*^*;Pten*^*+/−*^ and *Deptor*^*−/−*^*;Pten*^*+/−*^ littermate mice were subjected to IHC staining (**A**) or IB (**B**) with the indicated Abs. The staining quantification was determined by IHC scoring using an IRS system from three to seven random fields of tissue sections. * *p* < 0.05; ** *p* < 0.01. The band density was quantified using ImageJ and expressed as fold change relative to the control, with the control value arbitrarily set to 1. **C** A model for DEPTOR suppression of lymphomagenesis by promoting HUWE1-mediated EGFR polyubiquitination and proteasomal degradation via binding to HUWE1.

Given that KI of *Deptor*^*S275A*^ mutant suppresses lymphomagenesis via the reduction of EGFR levels and inactivation of both mTORC1/2 and MAPK-ERK pathways, we further determined whether DEPTOR disruption would induce EGFR levels and activate both pathways as well. Indeed, IHC staining of lymphomas from paired *Deptor*^*+/+*^*;Pten*^*+/−*^ and *Deptor*^*−/−*^*;Pten*^*+/−*^ mice showed a significantly higher staining of EGFR, p-ERK1/2, p-4E-BP1, and p-AKT (Fig. 7A), indicating the accumulation of EGFR levels and activation of the MAPK-ERK and mTORC1/2 signals in *Deptor*^*−/−*^*;Pten*^*+/−*^ lymphomas. Consistently, the elevated levels of EGFR, p-ERK1/2, p-4E-BP1, p-S6K1, and p-AKT were readily seen in *Deptor*^*−/−*^*;Pten*^*+/−*^ lymphomas, as compared to *Deptor*^*+/+*^*;Pten*^*+/−*^ lymphomas derived from three independent litters of mice (Fig. 7B). In addition, silencing of DEPTOR also induced EGFR levels and activates MAPK-ERK pathway in human B lymphoblastoid Raji cells (Fig. S7). Taken together, DEPTOR disruption induces EGFR levels and activates the MAPK-ERK and mTORC1/2 pathways, thereby promoting lymphomagenesis.

## Discussion

DEPTOR, a unique direct inhibitor of both mTORC1 and mTORC2, is often dysregulated in various cancers, including clear cell renal cell carcinoma tumor [25], colorectal cancer [26], esophageal squamous cell carcinoma [27], lung cancer [28], multiple myeloma [9], pancreatic cancer [29], and prostate cancer [11]. Its role is highly context-dependent, functioning either as a tumor suppressor or an oncogene, depending on the specific cellular or tissue environment [9, 10, 16, 30, 31]. Our previous studies demonstrated that DEPTOR inhibits prostate tumorigenesis by inactivating both mTORC1 and mTORC2 [11], and suppresses lung tumorigenesis by inhibiting EGFR-mTOR signaling [12]. Conversely, DEPTOR promotes the progression of ErbB2-positive breast cancer by stabilizing ErbB2 on the cell membrane [32]. In this study, we further strongly demonstrated that DEPTOR acts as a tumor suppressor in lymphomagenesis, supported by the following lines of evidence: (1) DEPTOR protein levels were decreased in human lymphoma tissues; (2) *Deptor* deletion accelerated lymphomagenesis induced by *Pten* inactivation and shortened lifespan in mice; (3) stablized DEPTOR via knock-in of degradation-resistant mutant inhibited *Pten* inactivation-driven lymphomagenesis in vivo; (4) DEPTOR inactivated EGFR-ERK1/2 signals by binding to HUWE1, enhancing the interaction between HUWE1 and EGFR, which promoted EGFR proteasomal degradation and subsequent inactivation of the ERK1/2 pathway.

It is well established that DEPTOR is degraded by the SCF^βTrCP^ ubiquitin ligase following its phosphorylation by S6K1 and RSK1 [13], or CK1α in conjunction with mTOR, which serves as a priming kinase [14, 15]. Numerous studies have demonstrated that the PI3K/AKT/mTOR pathway is aberrantly activated in lymphomas, including both Hodgkin and non-Hodgkin lymphomas [7, 33, 34]. Although RSK1 mRNA expression is relatively low in lymph nodes, its protein levels are markedly elevated in lymphoma tissues. Analysis of the Oncomine database further revealed that RSK1 is overexpressed across various lymphoma subtypes, with its expression levels negatively correlated with the survival of patients with DLBCL [35]. These findings suggest that RSK1 is hyperactivated in lymphoma tissues. In addition, CK1α has also been reported to exhibit hyperactivity in lymphomas [36]. Furthermore, βTrCP1 was found to be highly expressed in 46.5% (53/114) of DLBCL cases, with high βTrCP1 expression correlating with advanced tumor stages [37]. Collectively, these observations indicate that the kinases and ubiquitin ligase responsible for DEPTOR degradation are hyperactivated/upregulated in lymphoma, likely contributing to DEPTOR degradation. Moreover, the aberrantly activated mTOR pathway negatively regulates DEPTOR transcription [9]. Taken together, the upregulation of βTrCP1, alongside the activation of the mTOR pathway, RSK1, and CK1α, provides a plausible explanation for the observed downregulation of DEPTOR in human lymphoma tissues.

In lung cancer cells, DEPTOR binds to the kinase domain of EGFR, thereby inactivating EGFR signaling without altering EGFR levels [12]. To investigate whether DEPTOR similarly inhibits EGFR signaling in lymphomas, we analyzed p-EGFR levels in lymphomas from *Deptor* KI and KO mice. In *Deptor* KI mice, the reduction in the p-EGFR level was more pronounced than that of total EGFR (Fig. 2C). Conversely, in *Deptor* KO mice, the increase in p-EGFR levels was more significant than the change in total EGFR levels (Fig. 7B). These findings suggest that DEPTOR binding to EGFR may also inhibit its activity in lymphomas. Furthermore, DEPTOR promotes EGFR degradation via HUWE1 in lymphomas. We hypothesize that the differential protein levels of HUWE1 in the lung and lymph node tissues may account for the distinct outcomes of EGFR after binding to DEPTOR. Upon reviewing protein expression data across 45 organs, we observed that HUWE1 protein exhibits medium level in the lymph node but low level in the lung (Fig. S8A). Similarly, mRNA expression data revealed that HUWE1 is expressed at higher levels in 76 lymphoma cell lines compared to 232 lung cancer cell lines (Fig. S8B). Furthermore, IB analysis showed high HUWE1 levels in MYC-driven human Burkitt lymphoma and mouse diffuse high-grade blastic B cell lymphoma/leukemia [38]. Additionally, in lung cancer cells, DEPTOR depletion or overexpression significantly activates or inactivates EGFR as well as the AKT/mTOR pathway, a key downstream signaling pathway of EGFR. However, the ERK1/2 pathway, another major downstream pathway of EGFR, remains unaffected [12]. In contrast, in lymphomas, DEPTOR KO significantly activates, whereas DEPTOR KI inactivates both the AKT/mTOR and ERK1/2 pathways. This difference may be explained by the differential modes of DEPTOR regulation: in lung cancer, it appears to modulate EGFR phosphorylation, while in lymphomas, DEPTOR regulates both EGFR activity and its protein abundance.

The tumor-promoting role of EGFR is well-established across various human cancers [39], especially in lung cancer [40], colorectal cancer [41], and glioblastoma [42]. However, its role in lymphomagenesis remains less understood. EGFR mRNA levels are significantly upregulated in DLBCL, which is associated with markedly worse overall survival [43]. EGFR activation also induces ibrutinib resistance in DLBCL [44]. Importantly, *Egfr*^*L747P or S*^ mutant KI mice, which express a nuclear EGFR with a mutant nuclear export signal (NES) sequence, spontaneously develop B-cell lymphoma [45]. Furthermore, the COSMIC database indicates that somatic mutations in the EGFR NES region are present in patients with plasma cell lymphoma [46]. These findings suggest that EGFR may play a critical role in lymphomagenesis. Furthermore, as ERK1/2 activation stabilizes c-MYC [47] and Eμ-Myc transgenic mice spontaneously develop lymphomas, MYC stabilization by the EGFR-ERK axis may contribute to its role in promoting lymphomagenesis. In our study, we observed a decrease in EGFR levels and inactivation of ERK1/2 in lymphomas that developed in mice with knock-in overexpression of DEPTOR (Fig. 2). Conversely, in lymphomas from *Deptor* KO mice, we noted an increase in EGFR levels and activation of the ERK1/2 pathway (Fig. 7). These findings further underscore the critical role of the EGFR-ERK axis in the regulation of lymphomagenesis.

EGFR stability is essential for its proper function, with ubiquitination playing a key role in regulating both its stability and degradation. c-CBL, an E3 ubiquitin ligase, is a well-established negative regulator of EGFR, controlling its turnover [48, 49]. Additionally, several other E3 ligases, including SOCS4, SOCS5 [50–52], HUWE1 [21], FBXW2 [53], and FBXL2 [54], promote EGFR ubiquitination and degradation, while a few E3 ligases, such as SMURF2 [55] and RNF144A [56], ubiquitinate and stabilize EGFR. Using an affinity purification coupled with mass spectrometry, we identified a robust interaction between DEPTOR and HUWE1 (Fig. S4). HUWE1 is a large 482 kDa HECT E3 ligase [57] that has been implicated in promoting EGFR ubiquitination and degradation [21]. The crystal structure of *Nematocida* HUWE1 reveals that its N-terminal region forms a large, dynamic substrate-binding ring [22]. In binding assays with fragments of HUWE1, we found that DEPTOR binds to several fragments within this N-terminal region (Fig. 4D), aligning with the structure of the giant substrate-binding ring. These findings suggest that DEPTOR binds to EGFR to form a module that may facilitate its recognition and binding by the substrate-binding ring of HUWE1 (Fig. 4E). Given that DEPTOR is recognized by the substrate-binding ring of HUWE1, we sought to determine whether DEPTOR itself is a substrate of HUWE1. Knockdown of HUWE1 using two distinct siRNAs in lymphoma cells, including Raji, Jeko-1, and Daudi, did not lead to an increase in DEPTOR protein levels (Fig. S9A), nor did it extend the protein’s half-life (Fig. S9B). These findings suggest that DEPTOR may not be a substrate of HUWE1.

Given that DEPTOR inhibits EGFR-mTOR signaling by either inhibiting EGFR kinase activity [12] or inducing EGFR degradation, and simultaneously suppresses the kinase activities of both mTORC1 and mTORC2 [9], and that mTOR negatively regulates DEPTOR levels at both transcriptional [9] and post-translational levels [13–16], a feed-forward loop is established between low DEPTOR levels and high EGFR-mTOR signaling activity. Notably, DEPTOR protein levels are significantly diminished in human lymphoma tissues (Fig. 5). Therefore, restoring DEPTOR levels in lymphoma tissues would disrupt this feed-forward loop, leading to a dramatic suppression of EGFR-mTOR signaling and, subsequently, inhibiting tumor growth. Importantly, temsirolimus, a water-soluble derivative of rapamycin, was approved by the European Union in 2009 for the treatment of relapsed or refractory mantle cell lymphoma [58]. Rapamycin is a well-known mTORC1 inhibitor, while DEPTOR inhibits the broader EGFR-mTOR and EGFR-ERK1/2 signaling axes. Thus, our study implies that restoring DEPTOR levels through various approaches could represent a novel therapeutic strategy for lymphoma treatment.

In summary, our study highlights the pivotal role of DEPTOR in suppressing lymphomagenesis by inactivating EGFR-ERK1/2 signaling cascades. Under normal physiological conditions, DEPTOR enhances the binding of EGFR to HUWE1, an E3 ubiquitin ligase, thereby facilitating EGFR degradation, likely through the proteasome. This leads to the inactivation of ERK1/2, while DEPTOR simultaneously inhibits both mTORC1 and mTORC2, collectively suppressing lymphomagenesis (Fig. 7C). Conversely, in oncogenic settings such as partial PTEN inactivation, a decrease in DEPTOR levels stabilizes EGFR and alleviates mTORC1/2 inhibition, resulting in the activation of ERK1/2 and S6K1 to drive proliferation and growth, as well as AKT to enhance survival, ultimately promoting lymphomagenesis. Therefore, developing small molecules that restore DEPTOR levels to suppress EGFR-ERK1/2 and mTOR signaling could be a promising future strategy for lymphoma treatment.

## Material and Methods

### Cell lines and chemicals

HEK293, Raji, Jeko-1, and Daudi cells were obtained from ATCC. HEK293 cell was maintained in Dulbecco’s modified Eagle’s medium (DMEM) containing 10% (v/v) fetal bovine serum (FBS) and 1% penicillin/streptomycin (P/S). Raji, Jeko-1, and Daudi cells were cultured in RPMI-1640, containing 10% FBS and 1% P/S. Lymphoma-related primary cells were generated from lymphomas from mice and primary MEFs were isolated from day E13.5 embryos. They were maintained in DMEM containing 15% FBS, 1% P/S, and 0.1 mM MEM nonessential amino acids. MG132 (10012628, Cayman) and CHX (C7698, Sigma) were obtained from commercial sources.

### Generation of *Deptor*^*S275A*^ knock-in mice and PCR-based genotyping

The strategy for generation of *Deptor*^*S275A*^ knock-in (KI) mice is shown in Fig. S1A. Mouse embryonic stem (ES) cells were electroporated with a linearized vector that targeted exons 6 and 7 with the mutation of S275A at exon 6 and then selected by G418 for homologous integration clones. *Deptor*^*S275A*^-targeted ES cells were then injected into C57BL/6 blastocysts to generate chimeras, which were further bred with Black Swiss females to generate *Deptor*^*S275A-neo*^ mice. *Deptor*^*S275A*^ mice were obtained by crossing *Deptor*^*S275A-neo*^ mice with recombinase flippase (Flp) transgenic mice to remove the neomycin resistance cassette. The *Deptor* knockout (KO) mice were obtained and genotyped as previously described [11]. *MMTV-Cre* (JAX stock #003553), *Pten*^*fl/fl*^ (JAX stock #004597), and *Pten*^*+/−*^ (NCI Strain Code: 01XH3) mice were crossed with *Deptor* KI or KO mice to generate the mice of indicated genotype, respectively. For genotyping, genomic DNA was isolated from mouse tail tips as described [11]. The following primers were used to detect WT (174 bp) and KI (207 bp) alleles: 5’-CTA CGG GAC CTC ACC GAG AA-3’ and 5’-ATC CCA TCA CTT AGG GGT CAG A-3’. For the animal studies, all procedures were approved by Zhejiang University Laboratory Animal Center. Animal care was provided in accordance with the principles and procedures outlined in Chinese National Research Guide for the Care and Use of Laboratory Animals. Animal care and procedures were also approved by the University of Michigan Committee on Use and Care of Animals and performed in accordance with the principles and procedures outlined in the National Research Council Guide for the Care and Use of Laboratory Animals.

### Human tissue microarray and immunohistochemistry

Human tissue microarrays consisting of 45 cases of lymphoma and 25 normal lymph node tissues were obtained from Alenabio Biotech Company (Xi’an, China) and immunostained with anti-DEPTOR antibodies as previously described [11]. For immunohistochemistry (IHC), after deparaffinization, rehydration, antigen retrieval, and blocking, the microarrays or tissue sections were incubated overnight with primary antibodies at 4 °C. The antibodies were used as follows: DEPTOR (11816), p-4E-BP1 (T37/46) (2855), p-AKT (S473) (4060), Ki-67 (12202), and p-ERK1/2 (4376) (Cell Signaling Technology); EGFR (ab52894) (Abcam). After counterstaining with hematoxylin, the slides were then scanned by a section scanner (KFBIO, KF-FL-020). For quantitative evaluation, tissues in at least 5 random fields of each sample were photographed at 20× magnification and the photographs were then analyzed using an immunoreactive score (IRS) as the IHC scoring scheme. Stained tissues were classified into four groups according to the staining intensity: negative (0), weak (1), moderate (2) and strong (3). Depending on the percentage of positive cells, the proportion score of DEPTOR expression was classified as follows: 0, 0%; 1, ≤ 10%; 2, 11–50%; 3, 51–80%; and 4, ≥ 81%. The total scores were calculated by multiplying the proportion score by the intensity score.

### Immunoblotting and immunoprecipitaion

Lymphoma tissues or cells were lysed in lysis buffer with protease inhibitors and phosphatase inhibitors, which was followed by immunoblotting or immunoprecipitaion as previously described [59]. The following antibodies were used: p-4E-BP1 (T37/46) (2855), 4E-BP1 (9452), p-S6K1 (T389) (9234), p-AKT (S473) (4060), AKT (4691), p-EGFR (3777), EGFR (4267), DEPTOR (11816, for human samples), p-ERK1/2 (9101), ERK1/2 (4696), GβL (3274) (Cell Signaling Technology), DEPTOR (09-463, for mouse samples) (Millipore), DEPTOR (NBP1-49674, for mouse samples) (Novus), ATPA1 (14418-1-AP), HUWE1 (19430-1-AP) (Proteintech), EGFR (sc-373746), S6K1 (sc-230) (Santa Cruz), FLAG (F1804), ACTIN (A5441), Tubulin (T9026) (Sigma), bead-conjugated FLAG (A2220), and bead-conjugated HA (A2095) (Sigma).

### In vitro binding assay

The HA-tagged DEPTOR plasmid was transfected into HEK293 cells for 48 h, and then the cells were harvested for IP using HA-conjugated beads. The immunoprecipitated HA-DEPTOR was eluted from the beads by incubation with HA peptide (HY-P0239, MCE). For HA-tagged HUWE1 expression, the plasmid was transfected into HEK293 shDEPTOR cells for 48 h, followed by cell harvesting and lysis in buffer containing protease inhibitors. FLAG-tagged EGFR was pulled down using FLAG beads from HEK293 shDEPTOR cells transiently transfected with FLAG-EGFR plasmid. The beads were then incubated with lysates from HEK293 shDEPTOR cells expressing HA-HUWE1 and purified HA-DEPTOR for 4.5 h at 4 °C. The immunoprecipitates were washed four times with lysis buffer and analyzed by IB analysis.

### Subcellular fractionation

Plasma membrane and cytoplasmic fractions were isolated using the Minute™ Plasma Membrane Protein Isolation and Cell Fractionation Kit (SM-005, Invent) following the manufacturer’s instructions.

### Quantitative real-time reverse-transcription PCR (qRT-PCR)

Total RNA was extracted from homogenized lymphoma tissues using the TRIzol reagent (15596018, Invitrogen) and subsequently reverse-transcribed into cDNA with the PrimeScript RT reagent kit (RR037A, Takara). qRT-PCR was conducted using SYBR Premix Ex Taq (RR420A, Takara) on a CFX96 Real-Time PCR System (Bio-Rad). The relative mRNA expression levels of *Egfr* were quantified using *Gapdh* as the internal reference gene and calculated by the comparative Ct (2^-ΔΔCt^) method. The following primers were used for qRT-PCR analysis: 5’-ACG CAA GGA AGA CAT TCA CGA-3’ and 5’-TCA GAT TGC TCA CGG TGC G-3’ for *Deptor*; 5’-GAC TCC CCT CTT GAG TTC TCT GA-3’ and 5’-GGG AAC AGA TTG GTT TAC ATA TTC A-3’ for *Egfr*; 5’-GCC GCC TGG AGA AAC CTG CC-3’ and 5’-GGT GGA AGA GTG GGA GTT GC-3’ for *Gapdh*.

### Plasmid overexpression, siRNA silencing, and lentivirus-based shRNA silencing

The constructs expressing FLAG-tagged DEPTOR, DEP domain, PDZ domain, FLAG-tagged EGFR, and HA-tagged DEPTOR, along with the full-length and fragments of HUWE1, were previously described [12, 23]. HEK293 cells were transfected with the indicated plasmids using Lipofectamine 2000 (11668019, Invitrogen), and after 48 h, the cells were harvested for IP analysis. Lymphoma cells were transfected with indicated siRNAs in 60-mm dishes using Lipofectamine RNAiMAX (13778150, Invitrogen), and after 72 h, the cells were harvested for IB analysis. siCtrl: 5’-ATT GTA TGC GAT CGC AGA C-3’; siDEPTOR: 5’-GCC ATG ACA ATC GGA AAT CTA-3’; siHUWE1-1: 5’-AAT TGC TAT GTC TCT GGG ACA-3’; siHUWE1-2: 5’-CAC ACC AGC AAT GGC TGC CAG AAT T-3’.

For lentivirus-based shRNA silencing, the short hairpin sequences targeting DEPTOR (5’-GCC ATG ACA ATC GGA AAT CTA-3’) were cloned into the pLKO.1-puro vector. Packaging and infection of lentiviral shRNA viruses were performed as previously described [60]. In brief, 293 T cells were co-transfected with the pLKO.1 shRNA vector, psPAX2, and pMD2.G plasmids to generate lentiviruses. Virus-containing supernatants were collected, filtered through a 0.45 μm filter, and subsequently concentrated by centrifuging at 24,000 g for 4 h at 4 °C. The pelleted viruses were then resuspended in DMEM for further use.

### Quantitative proteomics analysis

Proteomics analysis was performed by Jingjie PTM BioLabs (Hangzhou, China) using 4D label-free proteomics technology. The workflow included trypsin digestion, liquid chromatography-mass spectrometry (LC-MS) analysis, database search, quantitative analysis, differential analysis, and functional enrichment analysis.

#### Trypsin digestion

Proteins were reduced with 5 mM DTT (30 min, 56 °C), alkylated with 11 mM iodoacetamide (15 min, room temperature, dark), and diluted with 100 mM TEAB to reduce the urea concentration (< 2 M). Proteins were digested with trypsin in two steps: 1:50 ratio (overnight) and 1:100 ratio (4 h). Peptides were desalted using a C18 SPE column.

#### LC-MS analysis

Peptides were dissolved in solvent A (0.1% formic acid, 2% acetonitrile) and separated using an EASY-nLC 1200 UPLC system with a gradient of solvent B (0.1% formic acid, 90% acetonitrile) at a 550 nl/min flow rate. Analysis was performed on an Orbitrap Exploris 480 mass spectrometer with nano-ESI ionization (2300 V). MS scans were set to a resolution of 60,000 (400–1200 m/z), and MS/MS scans at 15,000 resolution (fixed first mass: 110 m/z). 25 abundant precursors were fragmented using HCD (27% NCE) with 20-second dynamic exclusion.

#### Database search

MS/MS data were analyzed using Proteome Discoverer (v2.4.1.15) against the SwissProt human database with reverse decoys and contaminants. Trypsin was set as the cleavage enzyme (2 missed cleavages allowed). Modifications included carbamidomethylation (Cys) as fixed and oxidation (Met), deamidation (Asn, Gln), N-terminal acetylation, methionine loss, and methionine loss + acetylation as variable. Mass tolerances: 10 ppm (precursors) and 0.02 Da (fragments). FDR: <1% for proteins, peptides, and PSMs.

#### Quantitative analysis

Protein abundance data were normalized and corrected. For normalization across samples, protein abundances (*I*) were centralized and transformed into relative quantification values (*U*) for each sample using the formula: *U*_*ij*_ *= I*_*ij*_ */ Mean(I*_*j*_*)*. For systematic bias correction, the relative quantification values (*U*) were corrected using the median abundance value (*R*) across all samples using the formula: *R*_*ij*_ *= U*_*ij*_ */ Median(U*_*i*_*)*. Where *i* represents the sample and *j* represents the protein. These steps ensured accurate protein abundance values.

#### Differential analysis

Protein expression differences were analyzed pairwise. Fold change (FC) was calculated as: *FC*_*A/B,k*_ = *R*_*Ak*_ */ R*_*Bk*_. R represents the relative quantitative value of the protein. *k* denotes specific protein. Proteins with a FC > 1.5 were considered significantly up-regulated, while those with a FC < 1/1.5 were classified as significantly down-regulated.

#### Functional enrichment analysis

Using Fisher’s exact test, enriched functional terms were identified based on: fold enrichment > 1.5 and *p* value < 0.05.

### Statistical analysis

The IHC scores are presented as the mean ± SD, while the data of qRT-PCR from at least 3 independent biological experiments and mouse body weight are shown as mean ± SEM. The data were analyzed using GraphPad Prism 8 (GraphPad Software, Inc.). The Wilcoxon rank sum test and Student’s *t*-test were utilized to compare the expression between groups. Survival analysis was performed by Kaplan–Meier analysis and compared using the log-rank test. Statistical significance was determined as *p* < 0.05.

## Supplementary information

Supplementary information

Original IBs

## Data Availability

The authors declare that all data supporting the findings of this study are available with the article or from the corresponding author upon reasonable request. The original western blot data are provided in Supplemental Material.
