# Supplementary material for: DEPTOR suppresses lymphomagenesis by promoting EGFR degradation via HUWE1 E3 ligase
Source: Cell Death Differ. 2025 Apr 1;32(10):1820–32. doi: 10.1038/s41418-025-01497-5 (PMC12501038; doi:10.1038/s41418-025-01497-5)
Supplement: Supplementary file 1 — Supplementary information [file 41418_2025_1497_MOESM1_ESM.pdf]

Supplementary figure legends

Figure S1. Generation of *Deptor*$^{S275A/S275A}$ knock-in mice.

(A) The strategy used to target exons 6 and 7 of the *Deptor* allele. (B) PCR analysis of genomic DNA of tails from *Deptor*$^{+/+}$, *Deptor*$^{S275A/+}$ and *Deptor* $^{S275A/S275A}$ mice. (C) Gemonic DNA sequencing confirmed the mutation of S275A in *Deptor* $^{S275A/S275A}$ mice. (D–E) The primary MEFs and brain tissues from two litters of mice with indicated genotypes were harvested for immunoblotting (IB) to verify DEPTOR accumulation in *Deptor* $^{S275A/S275A}$ mice (top) and for qRT-PCR to assess DEPTOR mRNA levels (bottom). (F) DEPTOR$^{S275A/S275A}$ protein exhibits a significant prolongation of its half-life. Primary MEFs with indicated genotypes were treated with cycloheximide (CHX) for various time periods and then harvested for IB (left) with indicated antibodies (Abs). The band density was quantified by ImageJ, and the decay curves are shown (right). (G) *Deptor*$^{S275A/S275A}$ knock-in does not cause embryonic and postnatal death. The numbers of mice over 5 weeks of age with different *Deptor* genotypes were summarized. (H) The bodies of the littermate female and male mice with the indicated genotypes at the age of 3 weeks were weighed. Data are presented as mean ± SEM (D, E, H). For statistical analysis, significances were determined by Student's *t*-tests. *$p < 0.05$, **$p < 0.01$, ***$p < 0.001$, ns, not significant.

Figure S2. The lymphomas that develop in *Deptor*$^{S275A/S275A}$*;Pten*$^{+/–}$ and *Deptor*$^{–/–}$*;Pten*$^{+/–}$ mice comprise both T-cell and B-cell lymphomas.

The lymphomas from littermate mice with indicated genotypes were sectioned and then subjected to immunohistochemical (IHC) staining with indicated Abs. Scale bar: 30 µm.

Figure S3. DEPTOR^{S275A} promotes EGFR degradation via proteasome.

(A) DEPTOR^{S275A} reduces EGFR levels. The primary MEFs with indicated genotypes from two litters of mice were harvested for IB with indicated Abs. (B) Proteasome inhibitor MG132 restores the reduction of EGFR at the cell surface caused by DEPTOR^{S275A}. The primary MEFs with indicated genotypes were treated with MG132 for 6 h and then harvested for subcellular fractionation, followed by IB with the indicated Abs. The band density was quantified using ImageJ and expressed as fold change relative to the control, with the control value arbitrarily set to 1. D: DMSO; MG: MG132.

Figure S4. HUWE1 is a potential DEPTOR binding protein.

HEK293 cells transfected with indicated plasmids for 72 h were lysed and subjected to immunoprecipitation with FLAG beads. The beads were boiled for SDS-PAGE, followed by Commassie blue staining, and then the gels were subjected to mass spectrometry for identification of DEPTOR binding proteins.

Figure S5. The expression of DEPTOR is low in lymphiod neoplasm diffuse large B-cell lymphoma across TCGA tumors

The UALCAN database (https://ualcan.path.uab.edu/) was used to analyze the expression of DEPTOR across TCGA tumors. ACC: adrenocortical carcinoma; BLCA: bladder urothelial carcinoma; BRCA: breast invasive carcinoma; CESC: cervical squamous cell carcinoma; CHOL: cholangiocarcinoma; COAD: colon adenocarcinoma; DLBC: lymphiod neoplasm diffuse large B-cell lymphoma; ESCA: esophageal carcinoma; GBM: glioblastoma multiforme; HNSC: head and neck squamous cell carcinoma; KICH: kidney chromophobe; KIRC: kidney renal clear cell carcinoma; KIRP: kidney renal papillary cell carcinoma; LGG: brain lower grade glioma; OV: ovarian serous cystadenocarcinoma; MESO: mesothelioma; LIHC: liver hepatocellular carcinoma; LUAD: lung adenocarcinoma; LUSC: lung squamous cell carcinoma; PAAD: pancreatic adenocarcinoma; PRAD: prostate adenocarcinoma; PCPG: pheochromocytoma and paraganglioma; READ: rectum adenocacinoma; SARC: sarcoma; SKCM: skin cutaneous melanoma; LAML: acute myeloid leukemia; TGCT: testis germ cell tumors; THCA: thyroid carcinoma; THYM: thymoma; STAD: stomach adenocarcinomna; UCEC: uterine corpus endometrial carcinoma; UCS: uterine carcinosarcoma; UVM: uveal melanoma

Figure S6. *Deptor* disruption causes spontaneous lymphoma.

Whole body necropsy of a representative *Deptor*$^{-/-}$ female mouse at the age of 14 months (A). Arrows point to the enlarged cervical and mesenteric lymph nodes and thymus, respectively. The lymph node and thymus tissues from *Deptor*$^{-/-}$ mice were subjected to H&E staining and IHC staining with CD3 and B220 Abs as indicated (B). Scale bars: 100 μm (H&E), or 50 μm (CD3 or B220 staining).

Figure S7. DEPTOR knockdown induces EGFR levels and activates the MAPK-ERK signal.

Raji cells were transfected with indicated siRNA for 72 h and then harvested for IB with indicated Abs.

Figure S8. HUWE1 expression in human tissues.

The Human Protein Atlas data (https://www.proteinatlas.org) are used to show HUWE1 protein levels by IHC staining in various human tissues (A) and HUWE1 RNA expression in different cell lines (B).

Figure S9. DEPTOR may not be a substrate of HUWE1.

(A) HUWE1 knockdown does not increase DEPTOR protein levels. Raji, Jeko-1, and Daudi cells were transfected with indicated siRNA for 72 h, followed by being harvested for IB with indicated Abs. (B) HUWE1 knockdown does not stabilize DEPTOR. Raji cells transfected with indicated siRNA for 48 h were treated with CHX for various time periods, and then harvested for IB (top) with indicated Abs. The band densities were quantified using ImageJ, and the corresponding decay curves are shown (bottom).

Table S1. The differentially expressed proteins in the MAPK signaling pathway in lymphoma from *Deptor*$^{S275A/S275A}$*;Pten*$^{+/-}$ mice.

Xiong et al. Figure S1

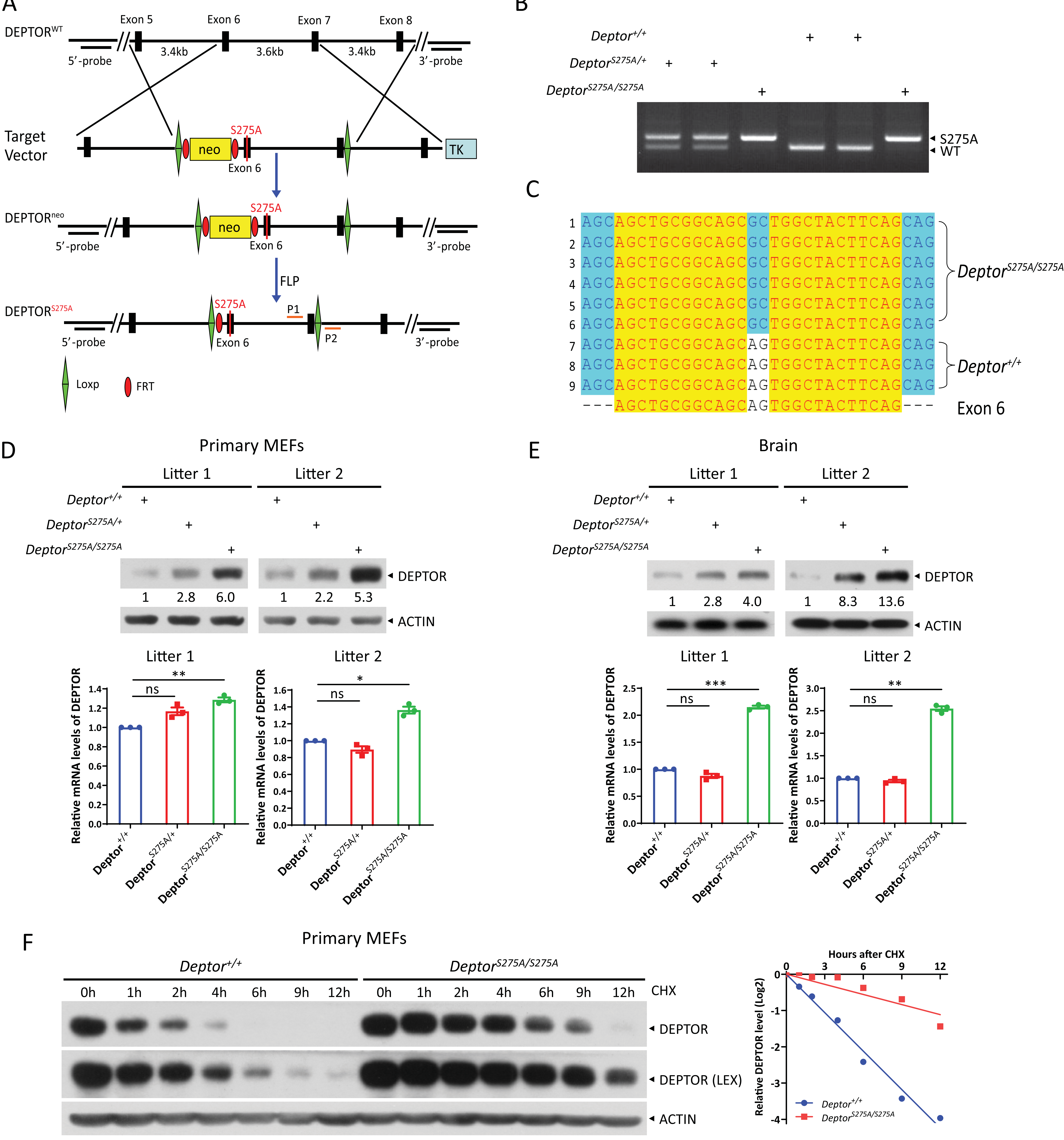

G

	$Deptor^{+/+}$	$Deptor^{S275A/+}$	$Deptor^{S275A/S275A}$	Total
No. of mice (>5wks)	45	99	44	188
Expected ratio	1/4	1/2	1/4	

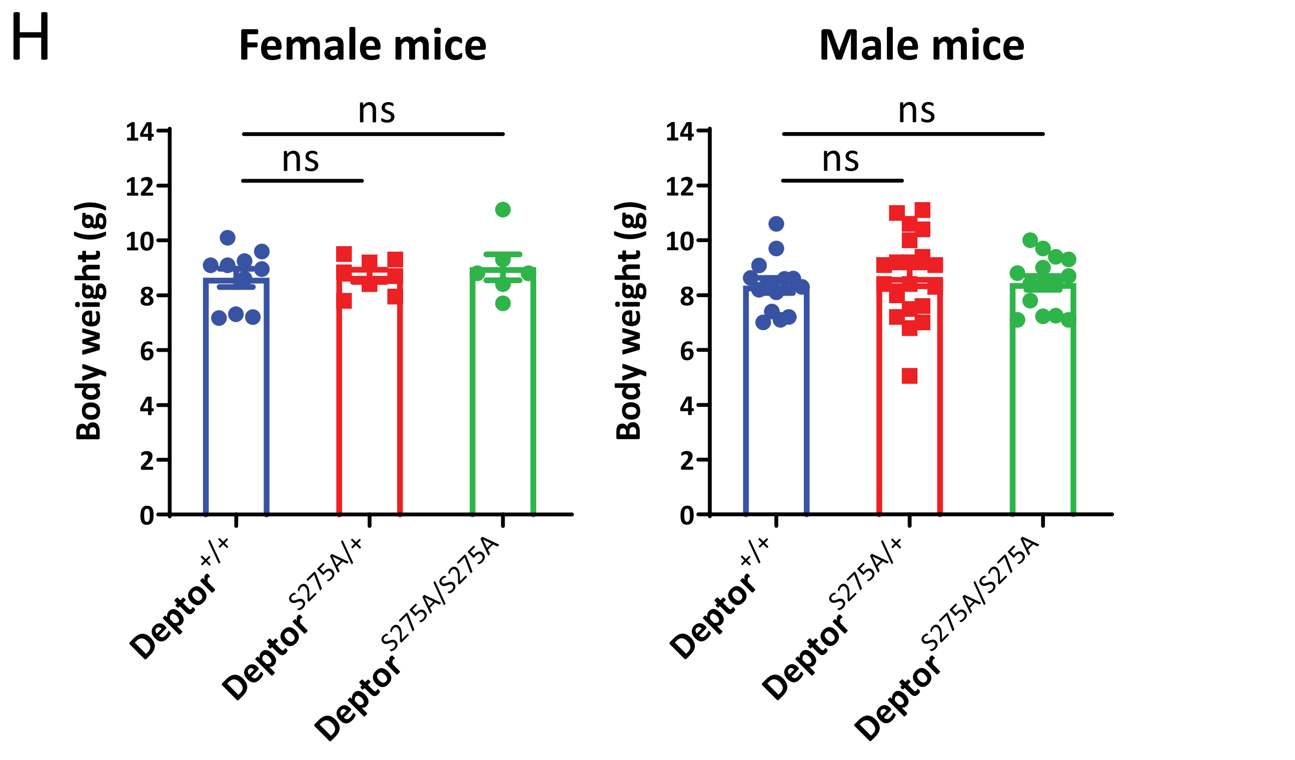

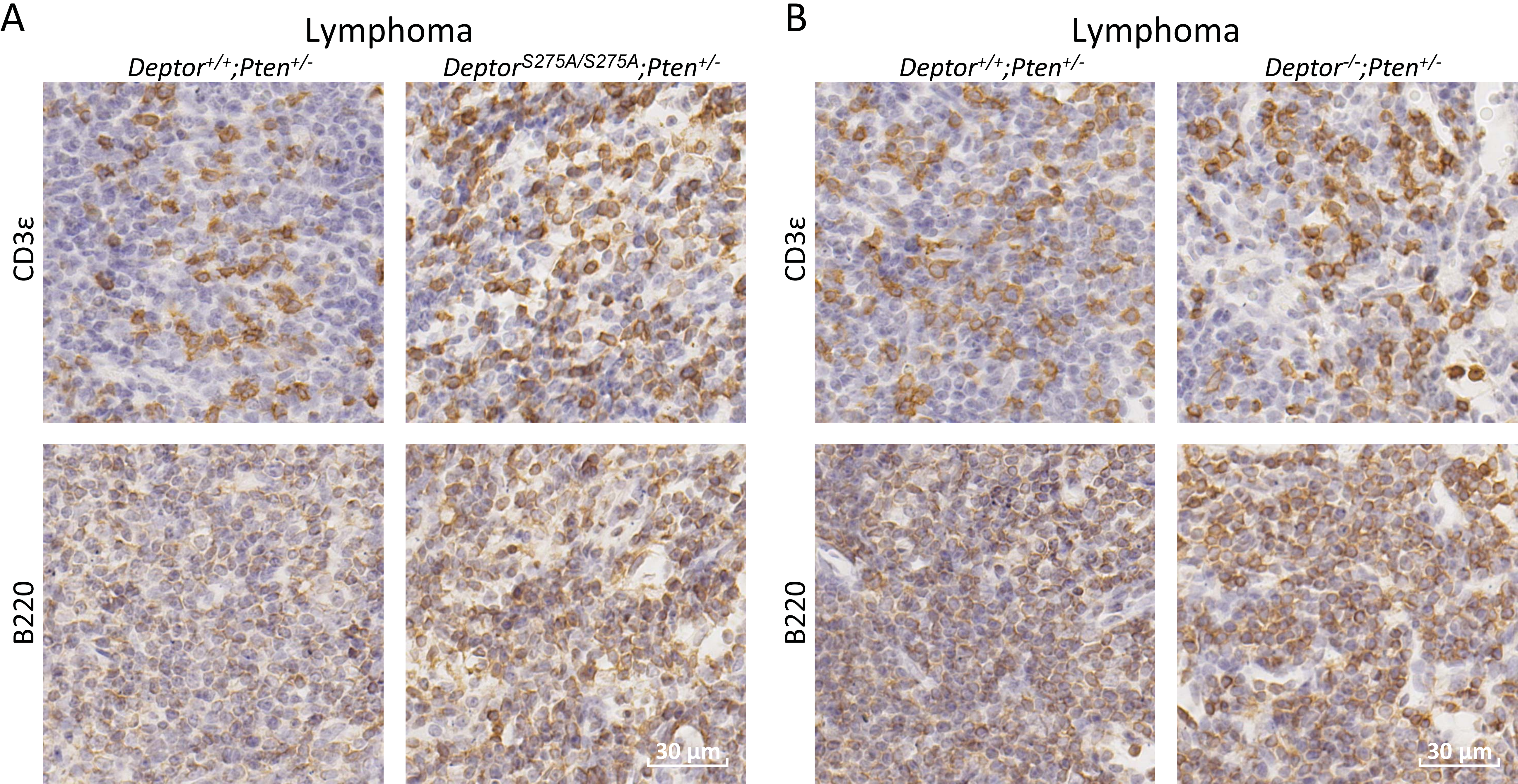
A
Lymphoma
Deptor+/+;Pten+/-
DeptorS275A/S275A;Pten+/-
CD3ε
B220
30 μm
B
Lymphoma
Deptor+/+;Pten+/-
Deptor-/-;Pten+/-
CD3ε
B220
30 μm

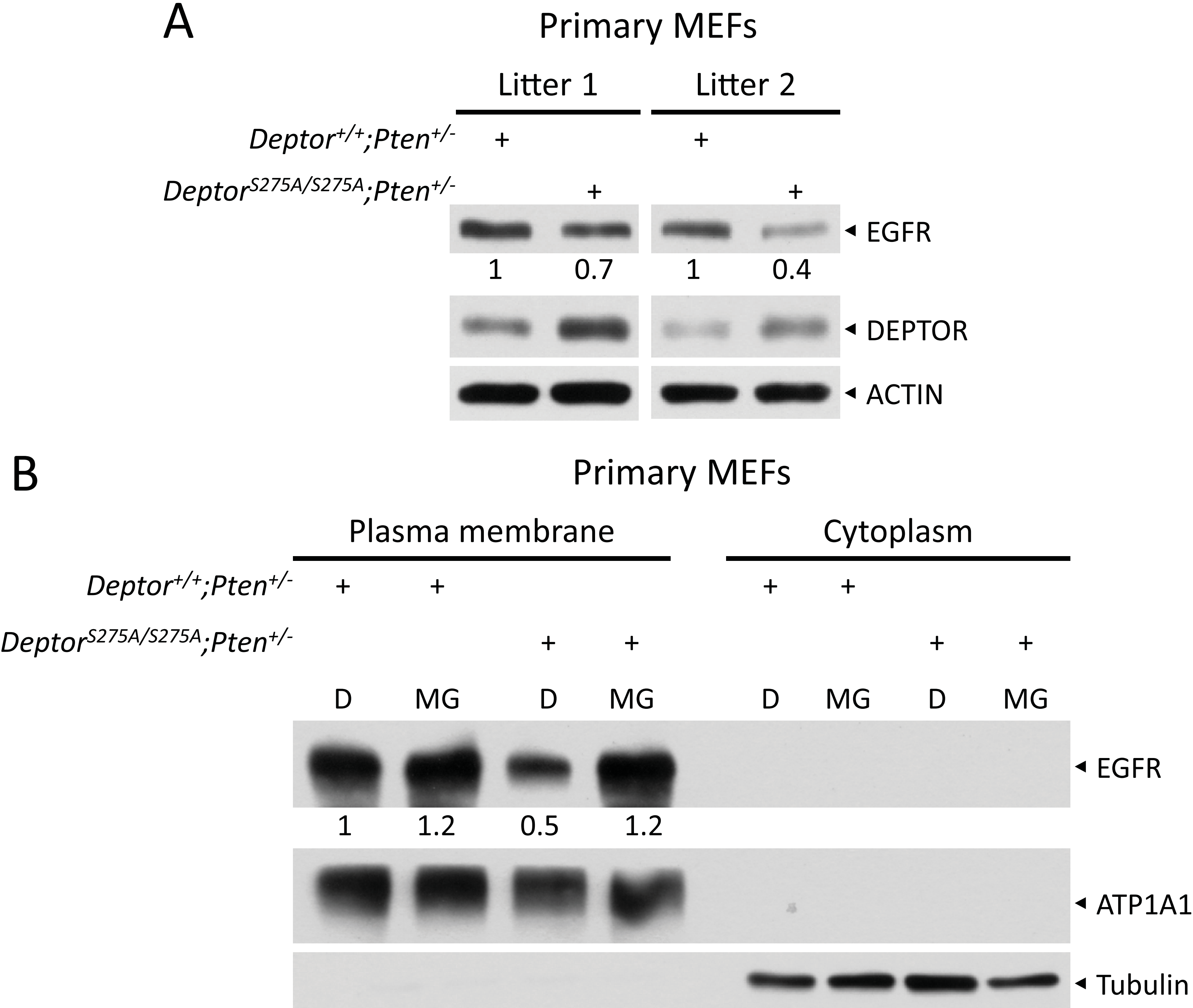
A
Primary MEFs
Litter 1
Litter 2
Deptor+/+;Pten+/-
DeptorS275A/S275A;Pten+/-
EGFR
1
0.7
1
0.4
DEPTOR
ACTIN
B
Primary MEFs
Plasma membrane
Cytoplasm
Deptor+/+;Pten+/-
DeptorS275A/S275A;Pten+/-
D
MG
D
MG
D
MG
D
MG
EGFR
1
1.2
0.5
1.2
ATP1A1
Tubulin

Xiong et al. Figure S4

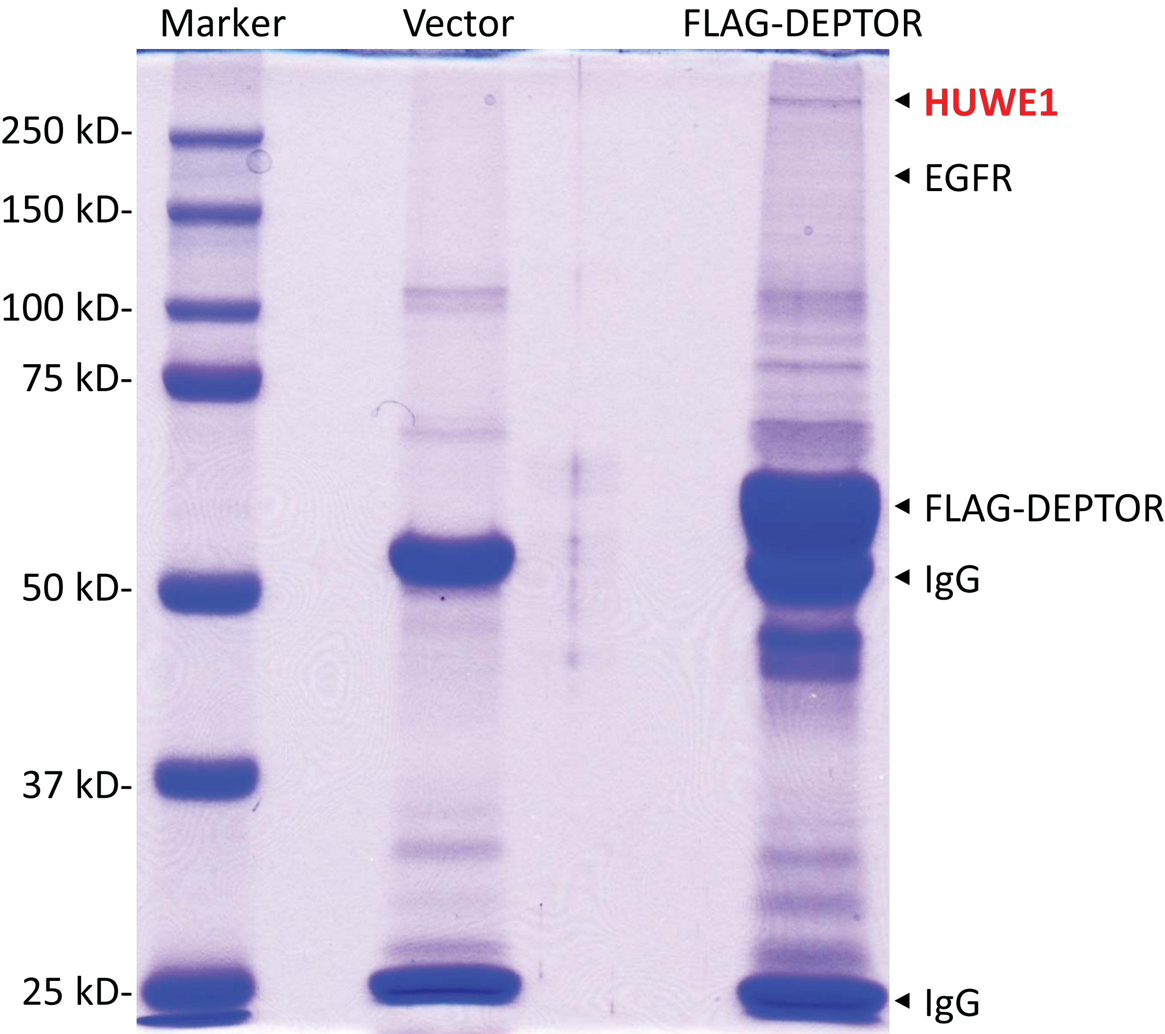

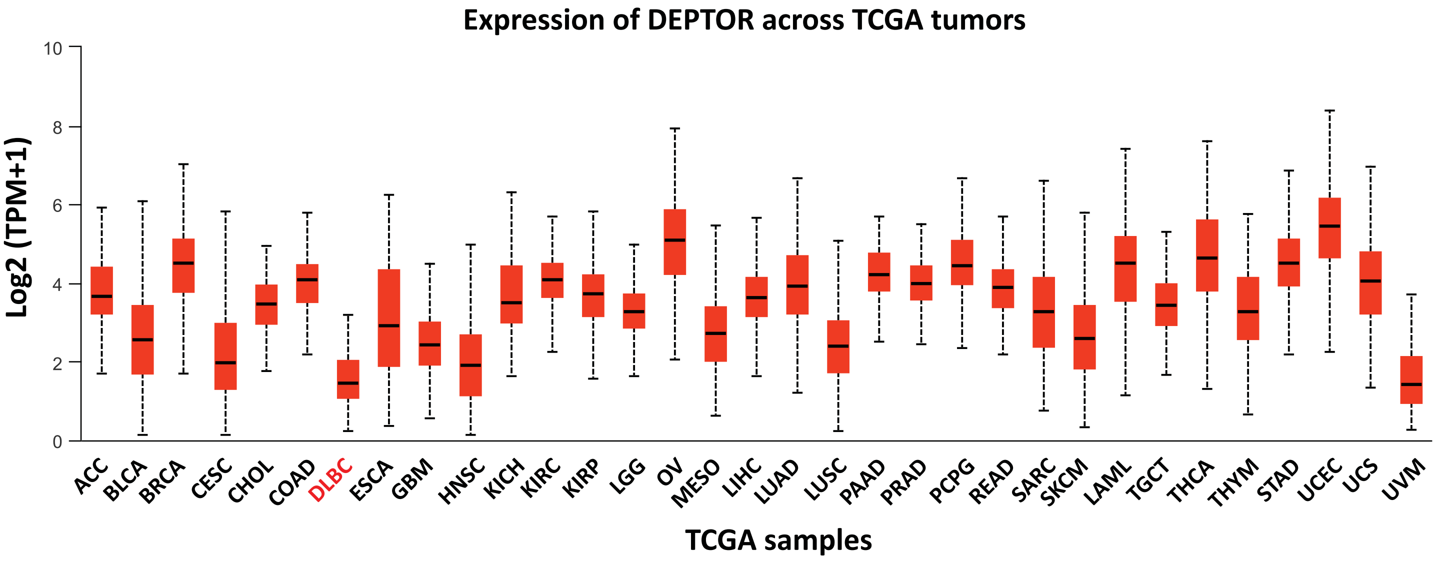
Expression of DEPTOR across TCGA tumors
Log2 (TPM+1)
10
8
6
4
2
0
ACC
BLCA
BRCA
CESC
CHOL
COAD
DLBC
ESCA
GBM
HNSC
KICH
KIRC
KIRP
LGG
OV
MESO
LIHC
LUAD
LUSC
PAAD
PRAD
PCPG
READ
SARC
SKCM
LAML
TGCT
THCA
THYM
STAD
UCEC
UCS
UVM
TCGA samples

Xiong et al. Figure S6

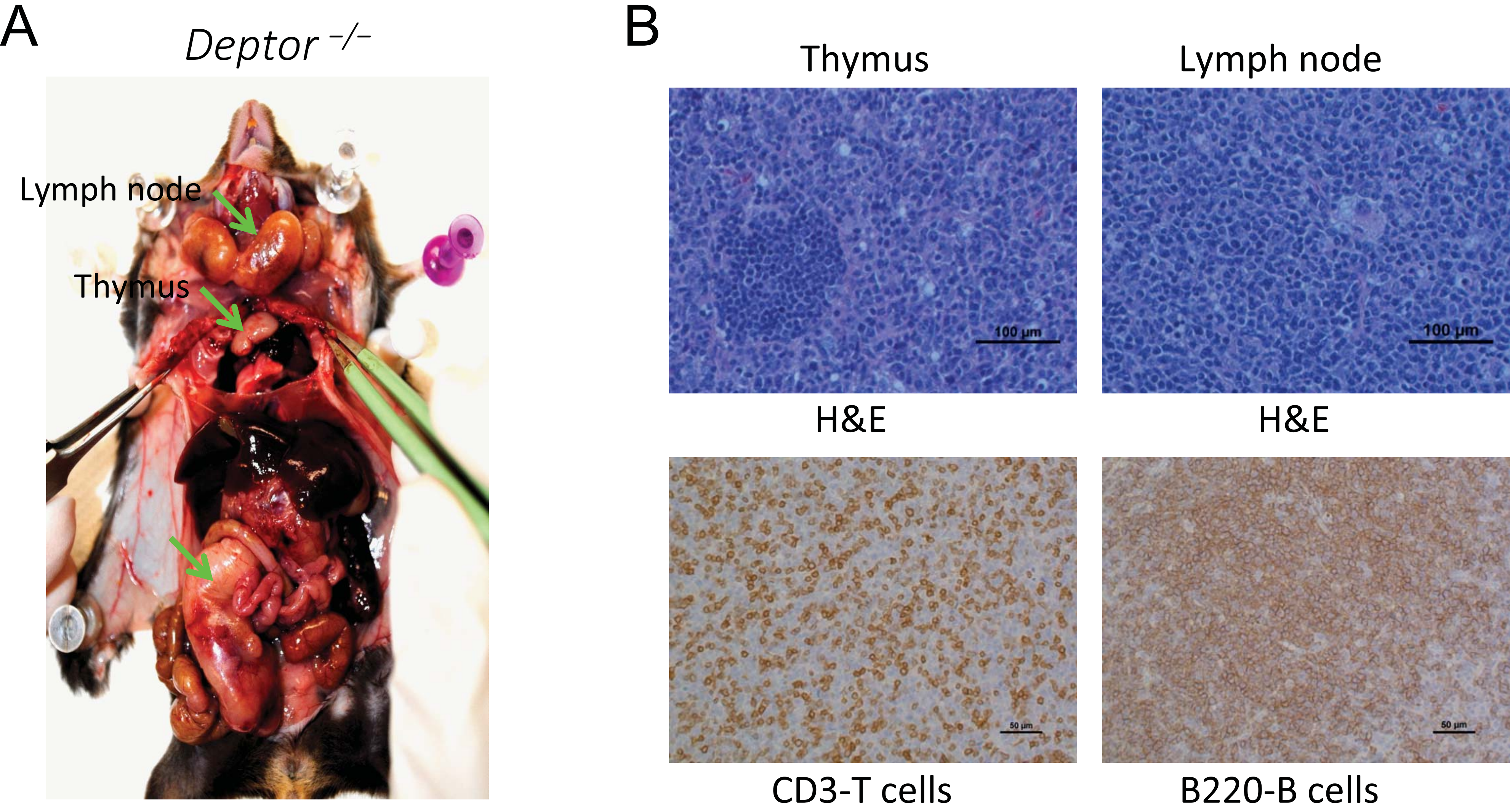

Xiong et al. Figure S7

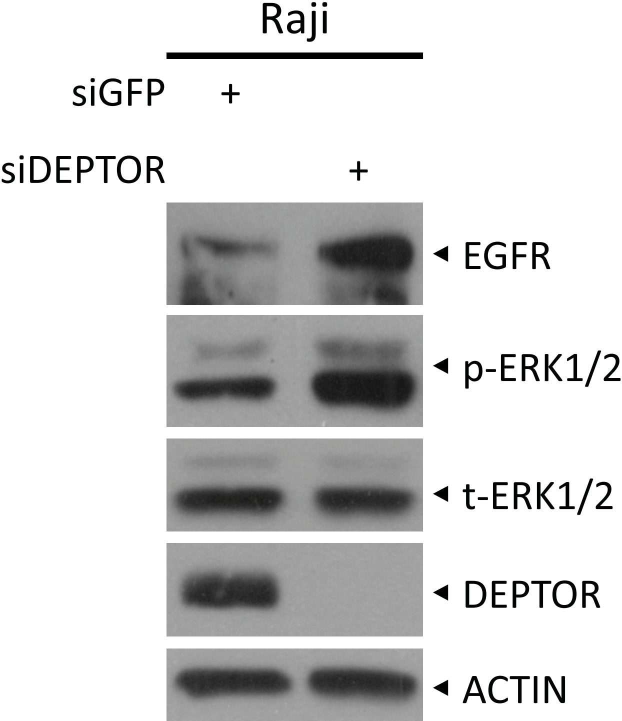

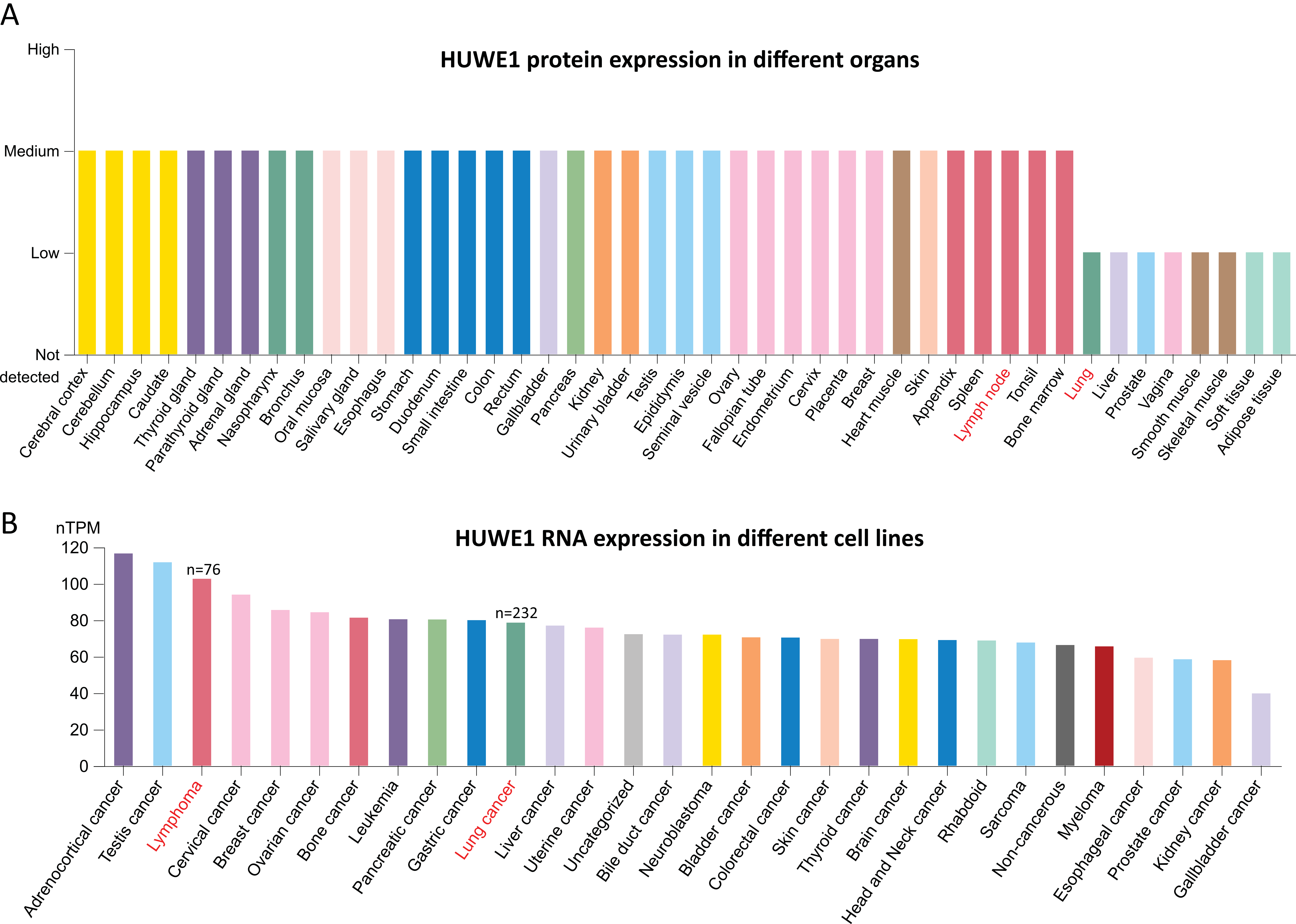
A
HUWE1 protein expression in different organs
High
Medium
Low
Not detected
Cerebral cortex
Cerebellum
Hippocampus
Caudate
Thyroid gland
Parathyroid gland
Adrenal gland
Nasopharynx
Bronchus
Oral mucosa
Salivary gland
Esophagus
Stomach
Duodenum
Small intestine
Colon
Rectum
Gallbladder
Pancreas
Kidney
Urinary bladder
Testis
Epididymis
Seminal vesicle
Ovary
Fallopian tube
Endometrium
Cervix
Placenta
Breast
Heart muscle
Skin
Appendix
Spleen
Lymph node
Tonsil
Bone marrow
Lung
Liver
Prostate
Vagina
Smooth muscle
Skeletal muscle
Soft tissue
Adipose tissue
B
HUWE1 RNA expression in different cell lines
nTPM
120
100
80
60
40
20
0
n=76
n=232
Adrenocortical cancer
Testis cancer
Lymphoma
Cervical cancer
Breast cancer
Ovarian cancer
Bone cancer
Leukemia
Pancreatic cancer
Gastric cancer
Lung cancer
Liver cancer
Uterine cancer
Uncategorized
Bile duct cancer
Neuroblastoma
Bladder cancer
Colorectal cancer
Skin cancer
Thyroid cancer
Brain cancer
Head and Neck cancer
Rhabdoid
Sarcoma
Non-cancerous
Myeloma
Esophageal cancer
Prostate cancer
Kidney cancer
Gallbladder cancer

Xiong et al. Figure S9

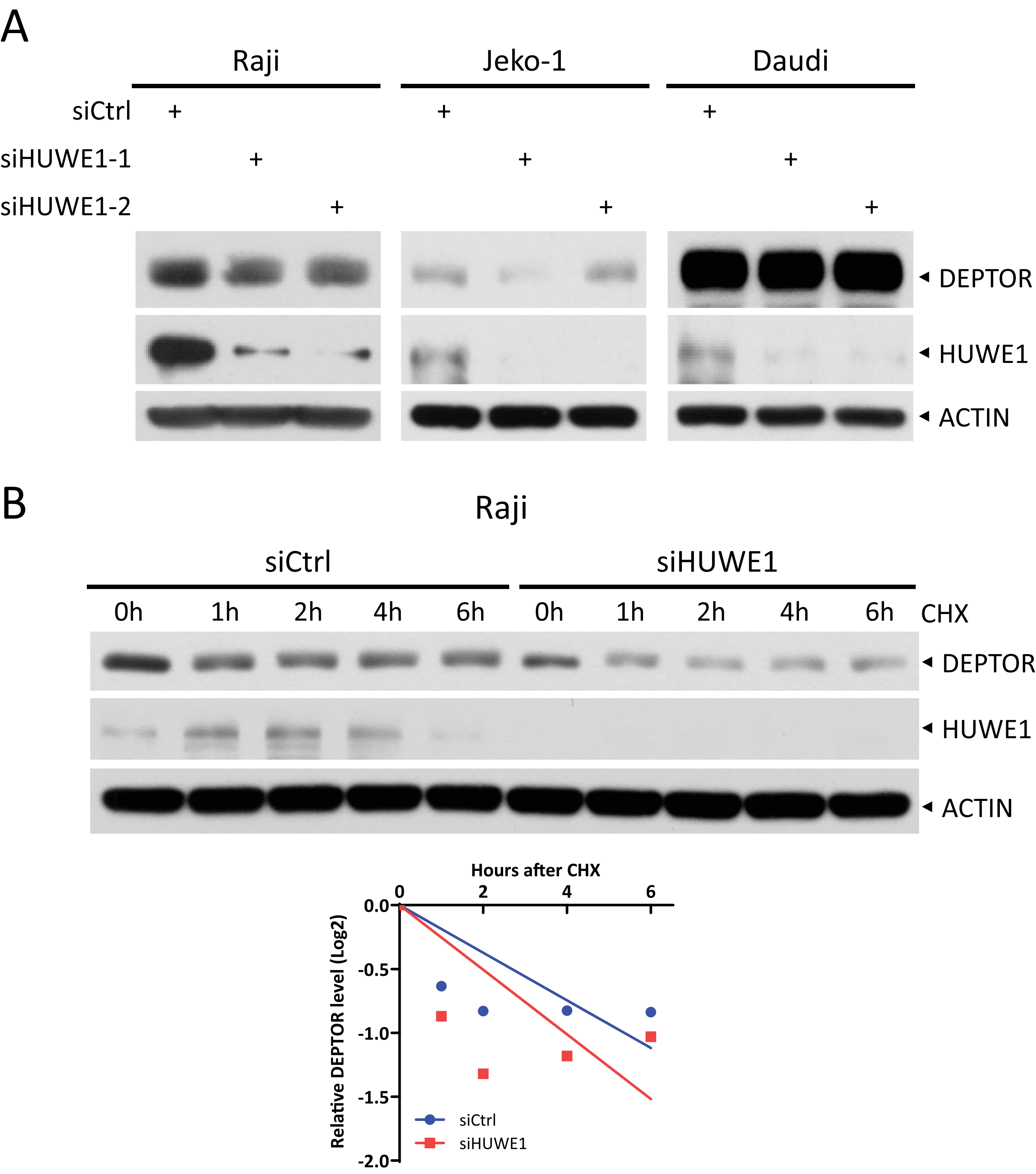

KEGG pathway	Fisher's exact test p value	Fold Enrichment	Protein accession	Gene name	KI/WT Ratio	Regulated Type	Coverage [%]	Peptides	PSM	Unique Peptides
map04010 MAPK signaling pathway	0.000369492	2.33	Q01279	Egfr	0.651	Down	16	14	30	14
map04010 MAPK signaling pathway	0.000369492	2.33	P31938	Map2k1	0.645	Down	39	13	76	9
map04010 MAPK signaling pathway	0.000369492	2.33	Q91YI4	Arrb2	0.323	Down	31	9	20	9
map04010 MAPK signaling pathway	0.000369492	2.33	Q9QZH6	Ecsit	0.579	Down	7	3	6	3
map04010 MAPK signaling pathway	0.000369492	2.33	P16627	Hspa1l	0.639	Down	16	9	71	1
map04010 MAPK signaling pathway	0.000369492	2.33	Q61084	Map3k3	0.646	Down	13	5	10	4
map04010 MAPK signaling pathway	0.000369492	2.33	O88942	Nfatc1	0.492	Down	5	3	7	3
map04010 MAPK signaling pathway	0.000369492	2.33	Q91Y86	Mapk8	0.587	Down	11	4	12	1
map04010 MAPK signaling pathway	0.000369492	2.33	P58069	Rasa2	0.66	Down	5	3	5	3
map04010 MAPK signaling pathway	0.000369492	2.33	Q99N57	Raf1	0.287	Down	9	5	12	3
map04010 MAPK signaling pathway	0.000369492	2.33	P28574	Max	0.62	Down	26	3	10	3
map04010 MAPK signaling pathway	0.000369492	2.33	P63328	Ppp3ca	0.661	Down	29	12	34	7
map04010 MAPK signaling pathway	0.000369492	2.33	P47809	Map2k4	0.619	Down	27	6	17	6
map04010 MAPK signaling pathway	0.000369492	2.33	P97820	Map4k4	0.17	Down	10	8	14	5
map04010 MAPK signaling pathway	0.000369492	2.33	O35613	Daxx	0.284	Down	4	2	7	2
map04010 MAPK signaling pathway	0.000369492	2.33	Q8BYC6	Taok3	0.517	Down	16	12	32	12
map04010 MAPK signaling pathway	0.000369492	2.33	Q8CF89	Tab1	0.61	Down	19	5	10	5
map04010 MAPK signaling pathway	0.000369492	2.33	P62071	Rras2	0.515	Down	34	6	24	4
map04010 MAPK signaling pathway	0.000369492	2.33	Q8CHG7	Rapgef2	0.463	Down	4	4	9	4
map04010 MAPK signaling pathway	0.000369492	2.33	Q5F2E8	Taok1	0.498	Down	6	4	8	4
