# Supplementary material for: DEPTOR suppresses lymphomagenesis by promoting EGFR degradation via HUWE1 E3 ligase
Source: Cell Death Differ. 2025 Apr 1;32(10):1820–32. doi: 10.1038/s41418-025-01497-5 (PMC12501038; doi:10.1038/s41418-025-01497-5)
Supplement: Supplementary file 2 — Original IBs [file 41418_2025_1497_MOESM2_ESM.pdf]

Figure 1F

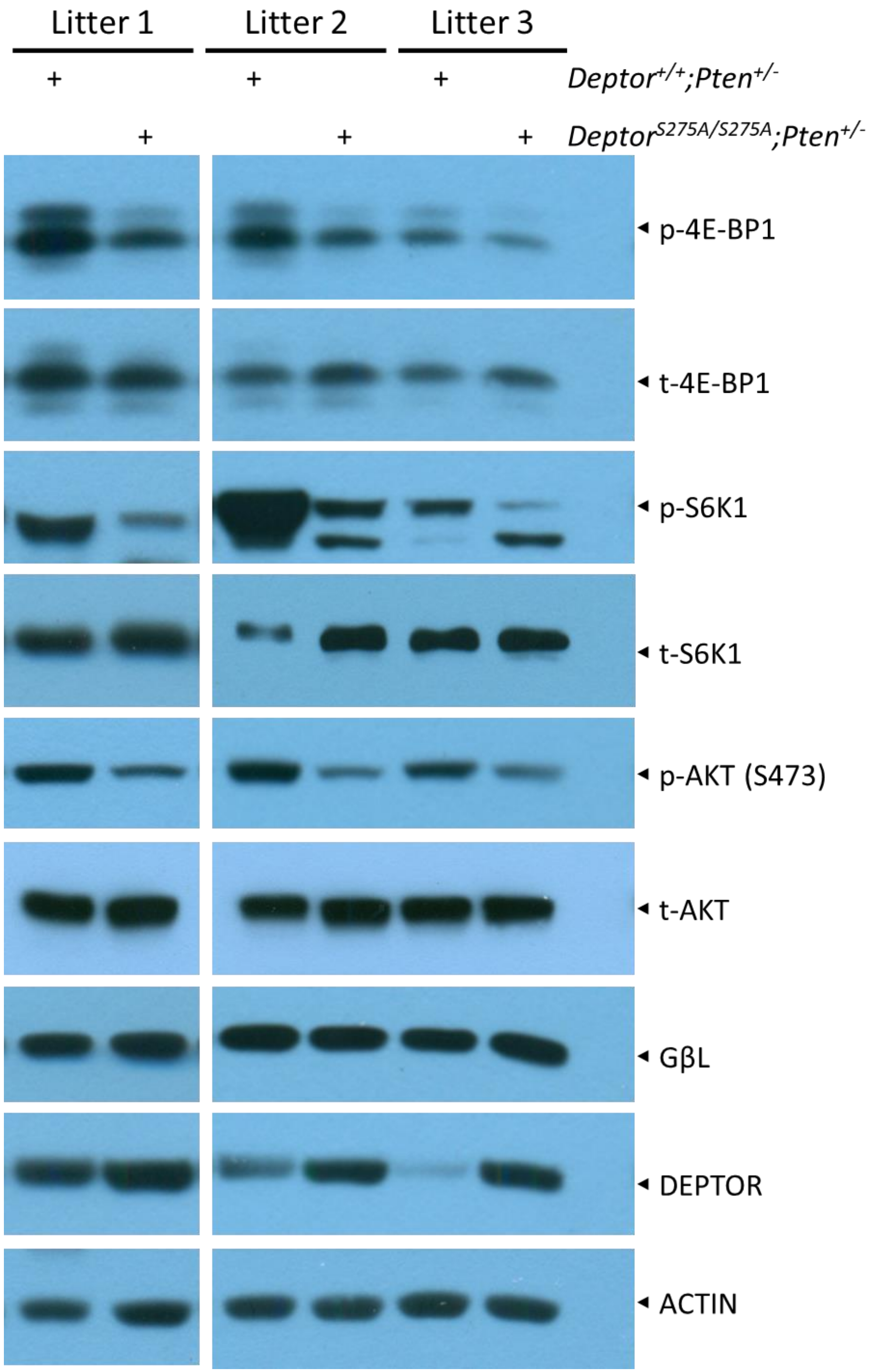

Litter 1
Litter 2
Litter 3
+ + + Deptor+/+;Pten+/-
+ + + DeptorS275A/S275A;Pten+/-
p-4E-BP1
t-4E-BP1
p-S6K1
t-S6K1
p-AKT (S473)
t-AKT
GβL
DEPTOR
ACTIN

Figure 2C

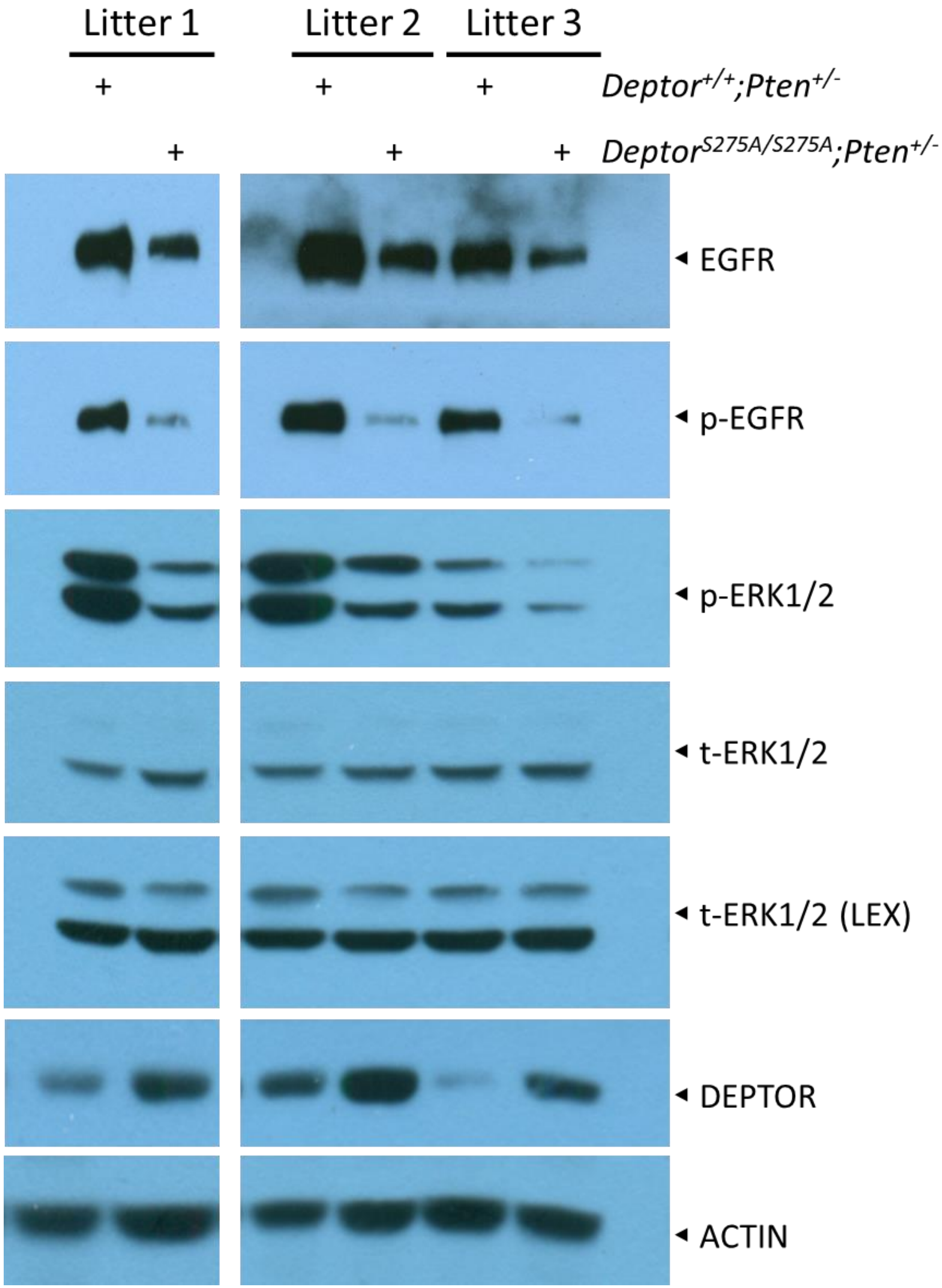

Litter 1
Litter 2
Litter 3
+ + + Deptor+/+;Pten+/-
+ + + DeptorS275A/S275A;Pten+/-
EGFR
p-EGFR
p-ERK1/2
t-ERK1/2
t-ERK1/2 (LEX)
DEPTOR
ACTIN

Figure 3A

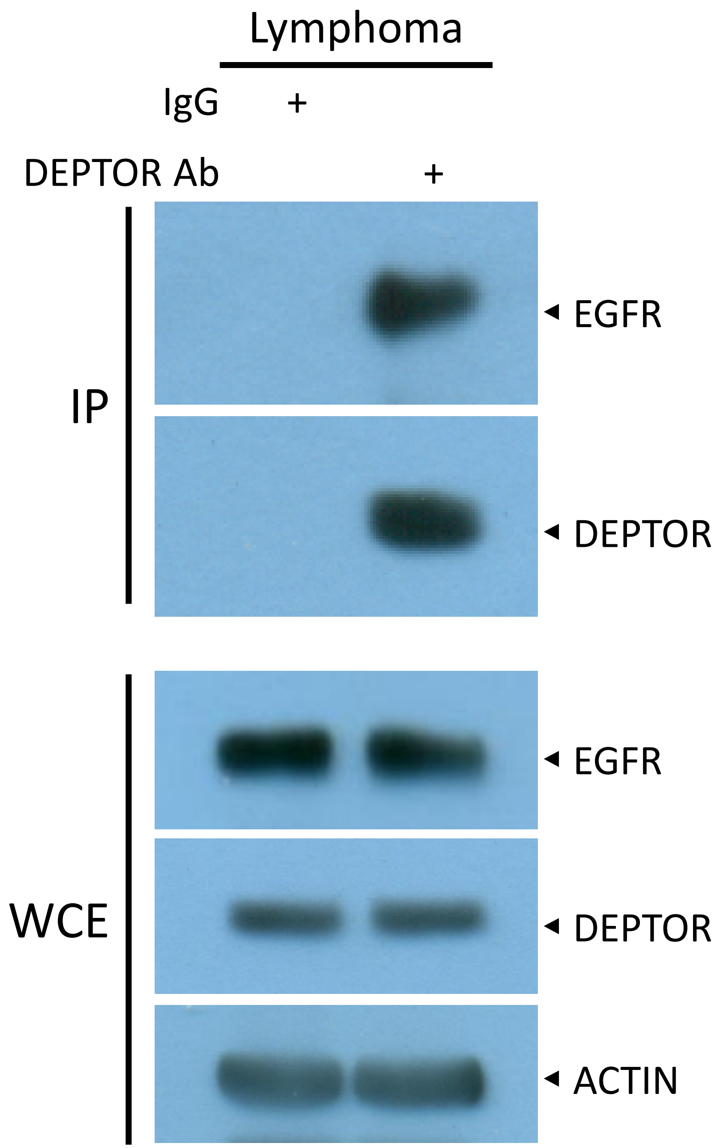

Figure 3C

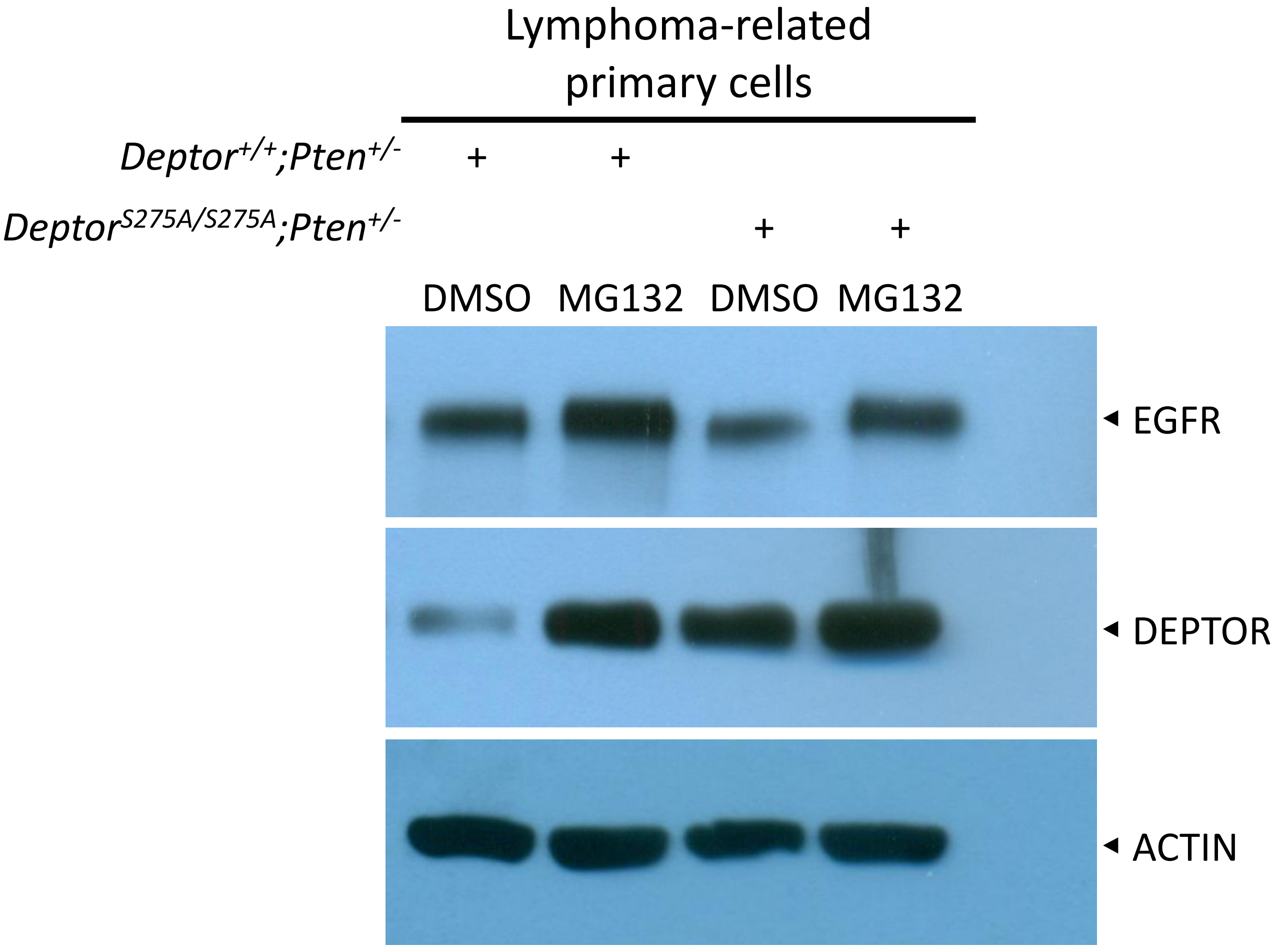

Lymphoma-related primary cells
Deptor+/+;Pten+/-
DeptorS275A/S275A;Pten+/-
+
+
+
+
DMSO
MG132
DMSO
MG132
EGFR
DEPTOR
ACTIN

Figure 3D

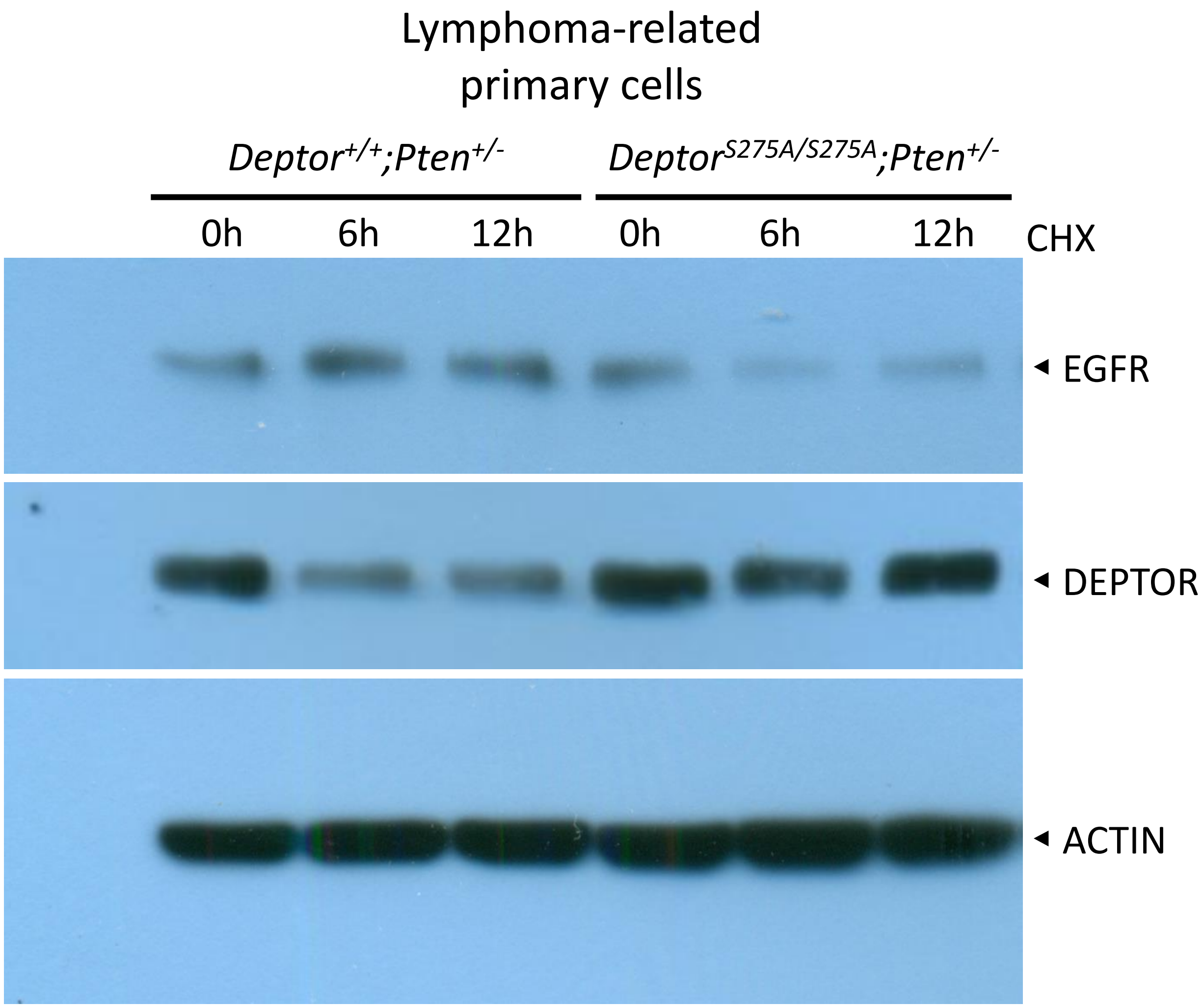

Lymphoma-related primary cells
Deptor+/+;Pten+/-
DeptorS275A/S275A;Pten+/-
0h
6h
12h
0h
6h
12h
CHX
EGFR
DEPTOR
ACTIN

Figure 4A

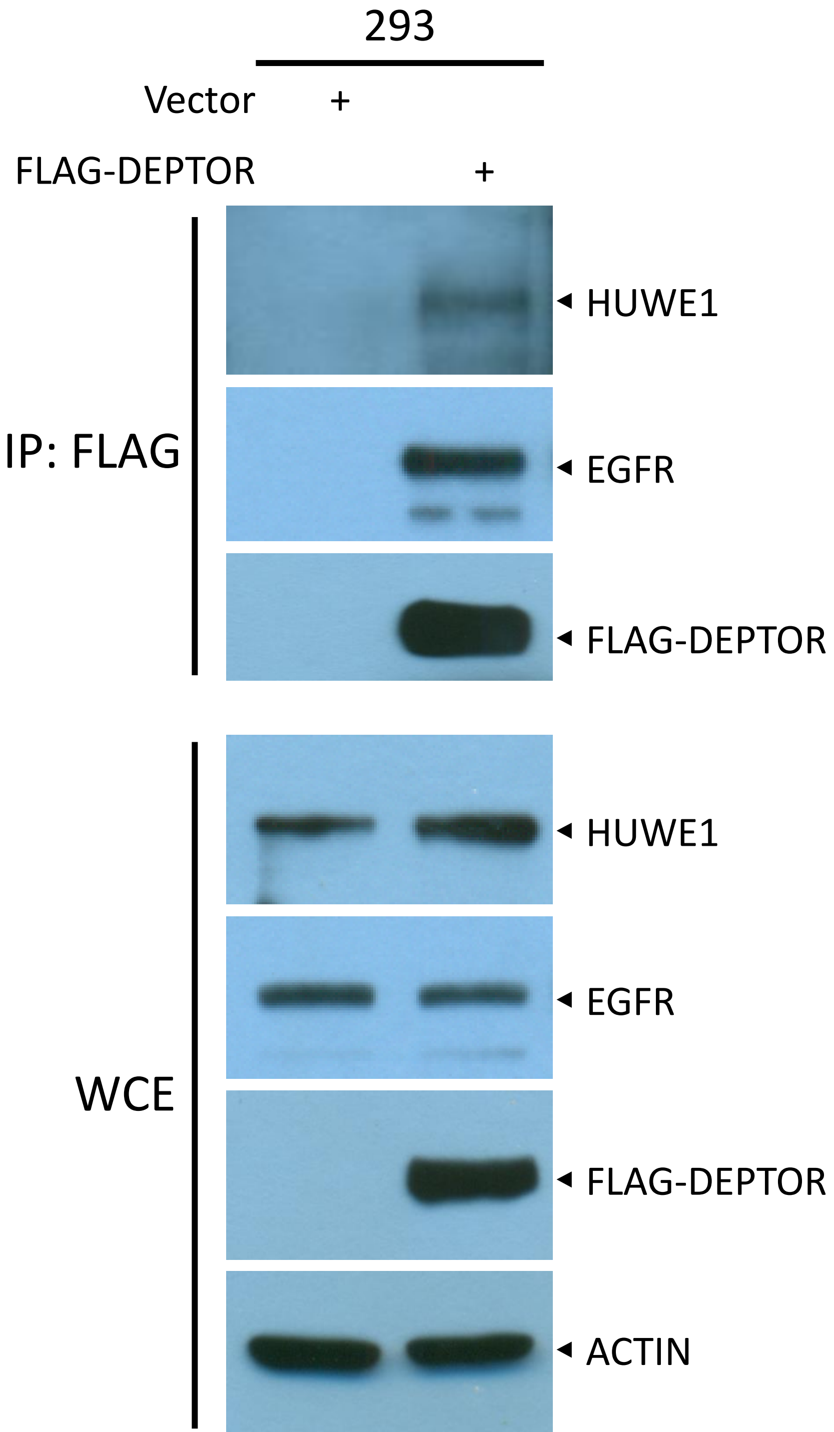
293
Vector
+
FLAG-DEPTOR
+
IP: FLAG
HUWE1
EGFR
FLAG-DEPTOR
WCE
HUWE1
EGFR
FLAG-DEPTOR
ACTIN

Figure 4B

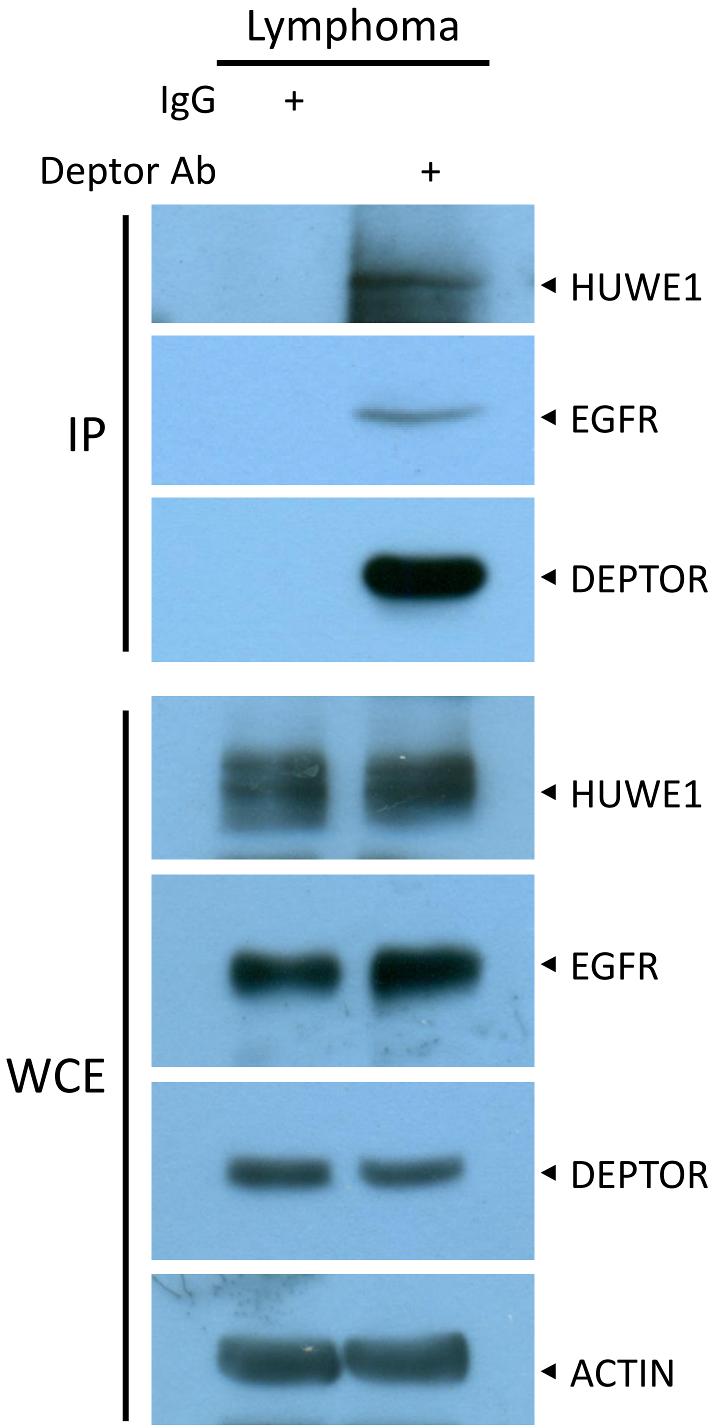

Lymphoma
IgG
+
Deptor Ab
+
IP
HUWE1
EGFR
DEPTOR
WCE
HUWE1
EGFR
DEPTOR
ACTIN

Figure 4C

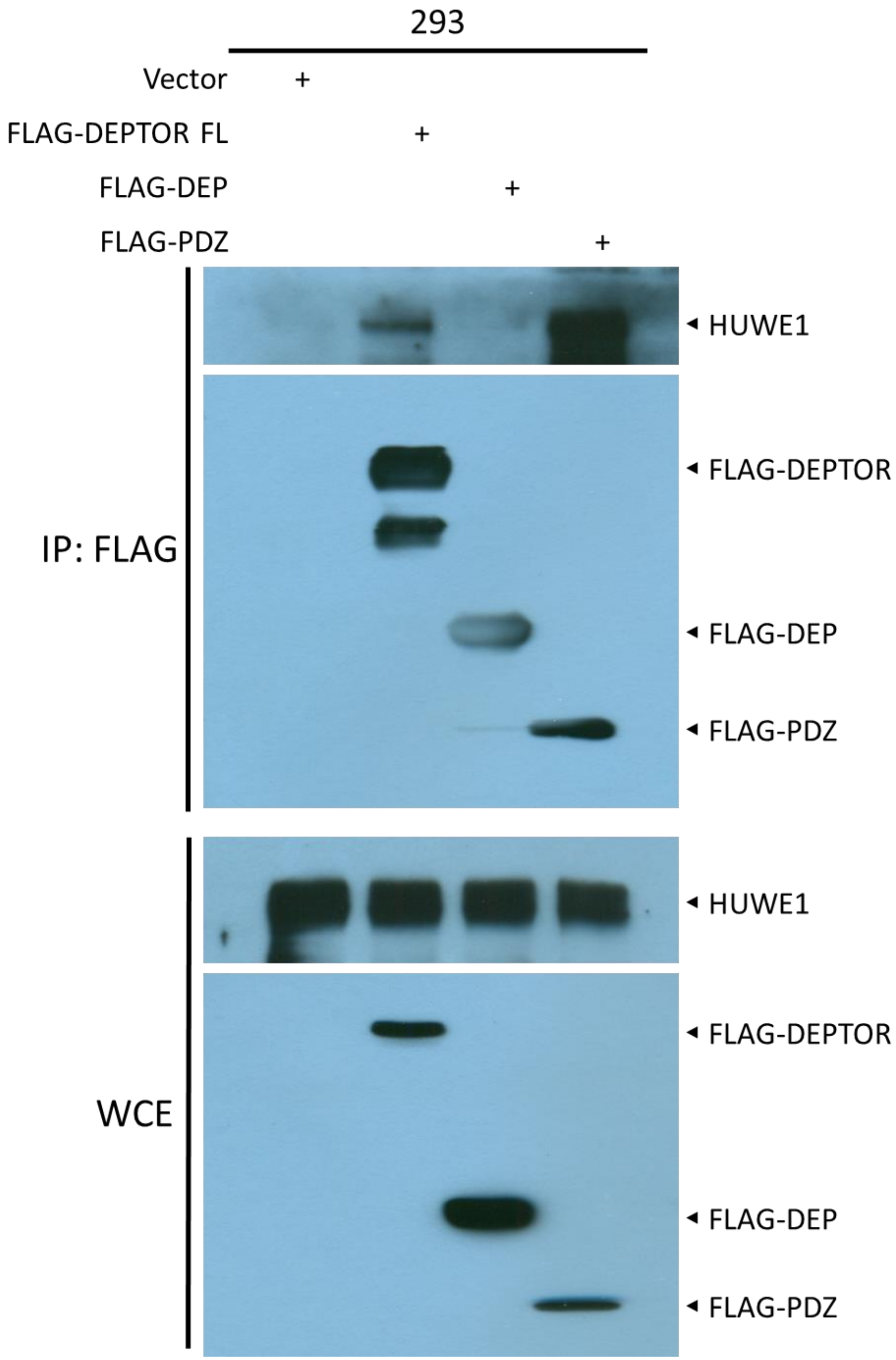

293
Vector +
FLAG-DEPTOR FL +
FLAG-DEP +
FLAG-PDZ +
IP: FLAG
HUWE1
FLAG-DEPTOR
FLAG-DEP
FLAG-PDZ
WCE
HUWE1
FLAG-DEPTOR
FLAG-DEP
FLAG-PDZ

Figure 4D

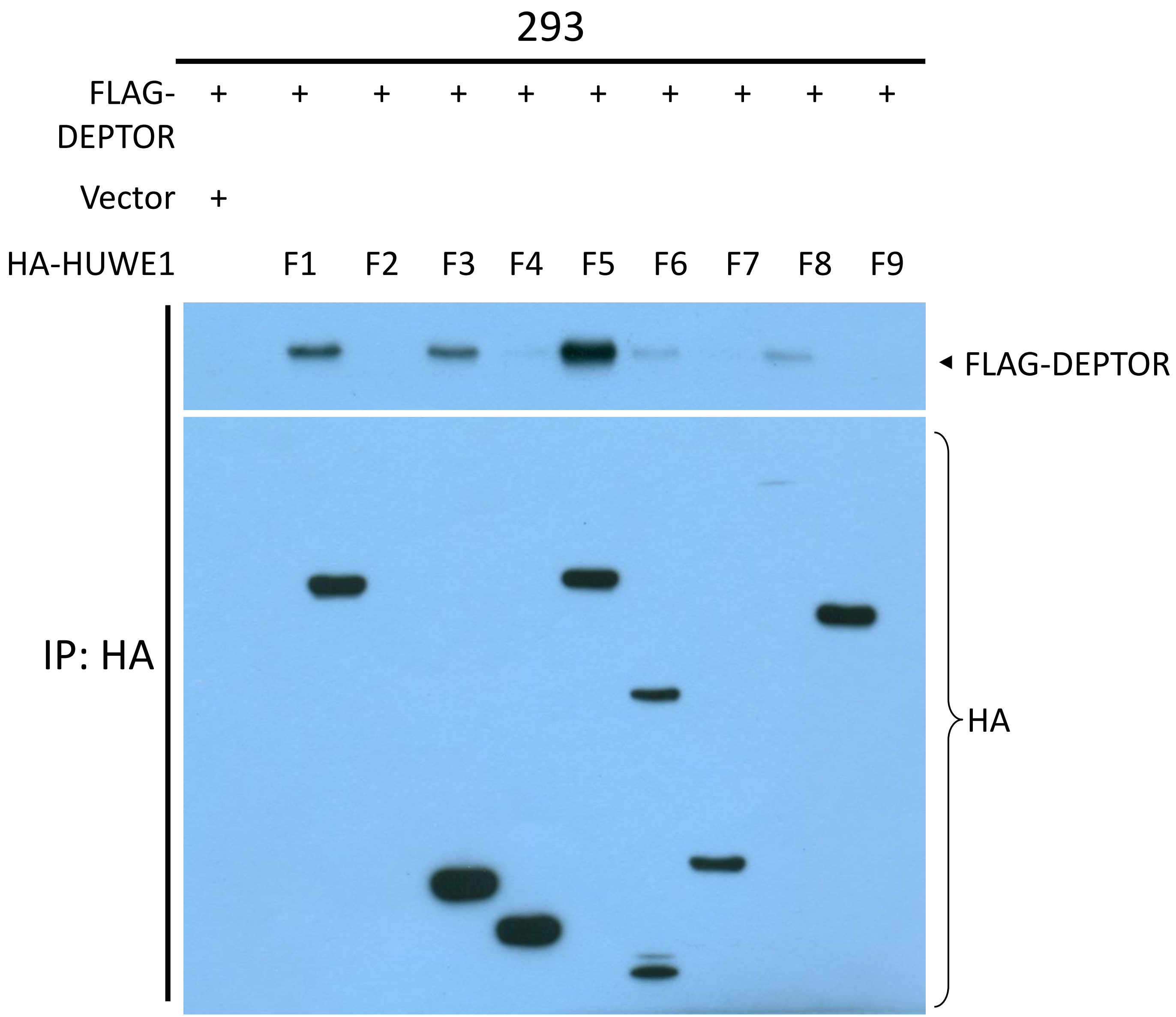
293
FLAG-DEPTOR + + + + + + + + + +
Vector +
HA-HUWE1 F1 F2 F3 F4 F5 F6 F7 F8 F9
IP: HA
FLAG-DEPTOR
HA

Figure 4D

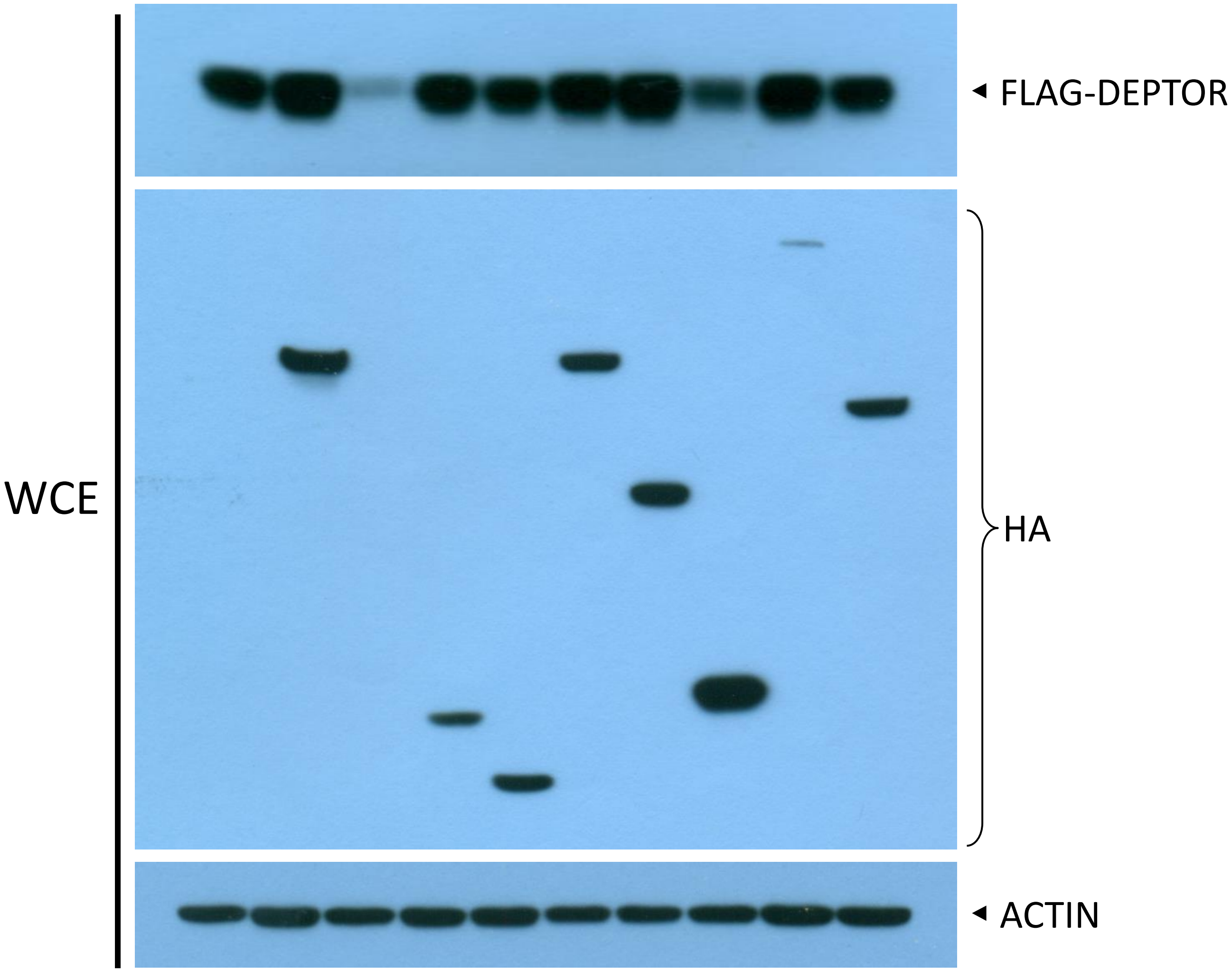

Figure 4F

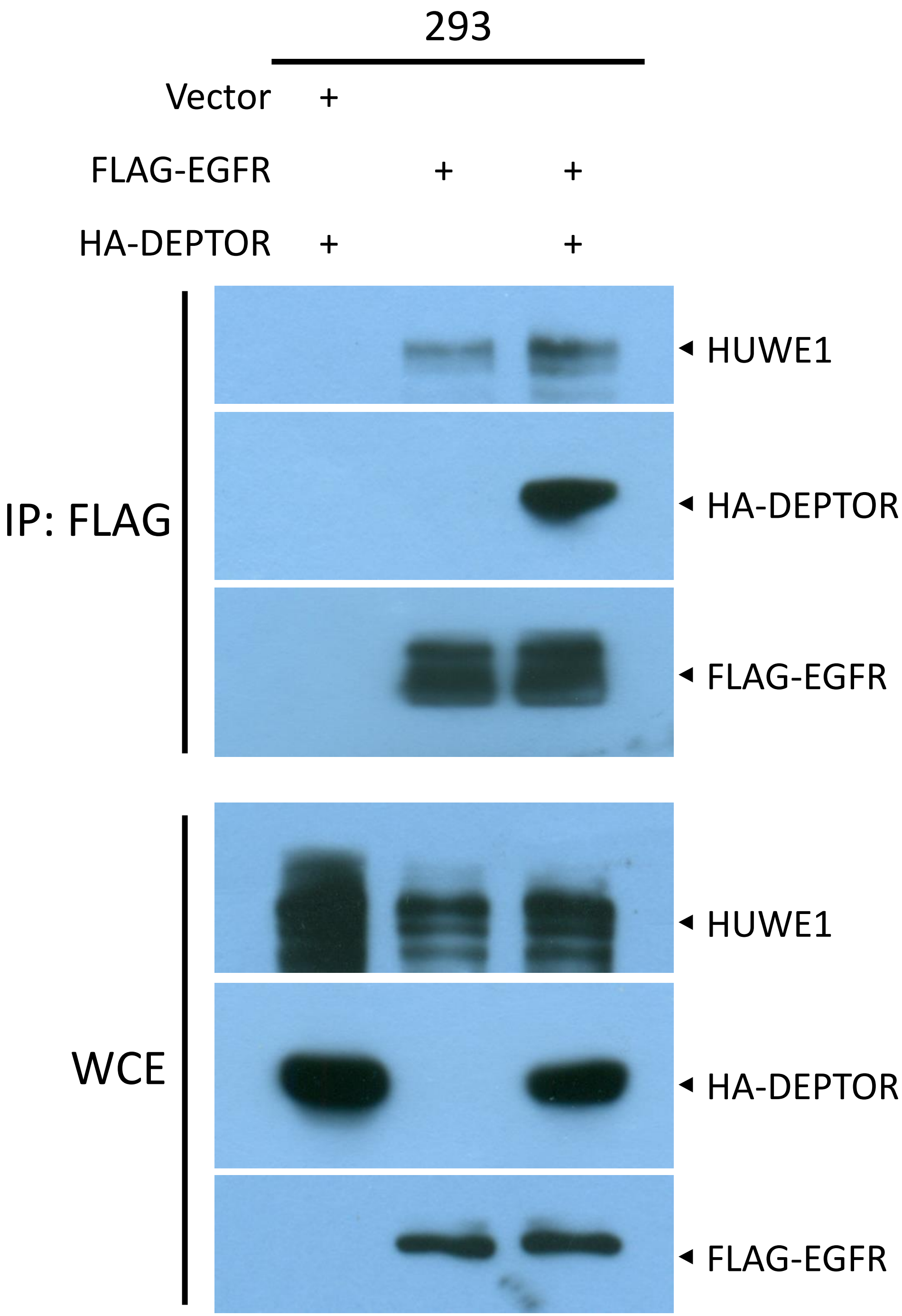

293
Vector +
FLAG-EGFR + +
HA-DEPTOR + +
IP: FLAG
HUWE1
HA-DEPTOR
FLAG-EGFR
WCE
HUWE1
HA-DEPTOR
FLAG-EGFR

Figure 4G

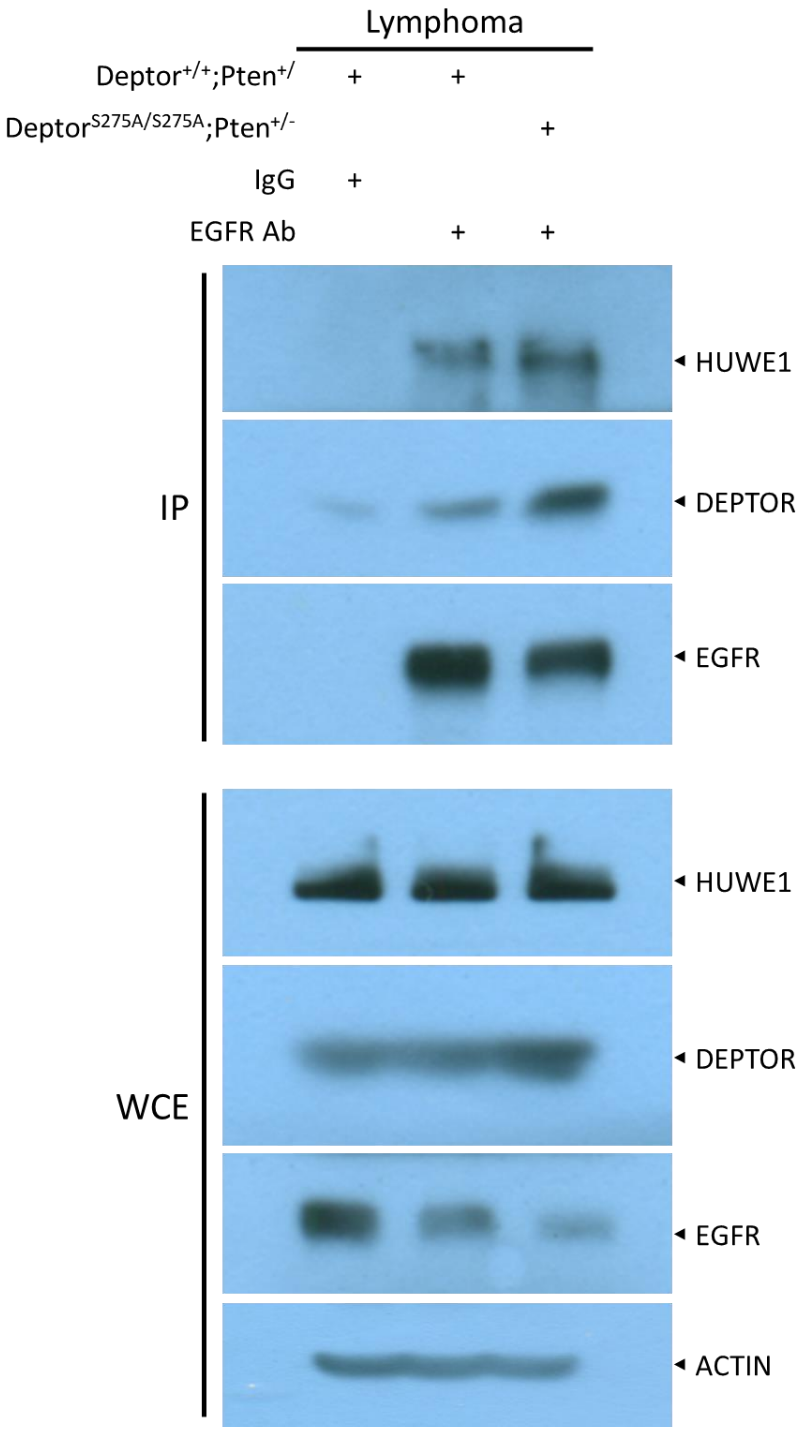
Lymphoma
Deptor$^{+/+}$;Pten$^{+/}$ + +
Deptor$^{S275A/S275A}$;Pten$^{+/-}$ +
IgG +
EGFR Ab + +
IP
HUWE1
DEPTOR
EGFR
WCE
HUWE1
DEPTOR
EGFR
ACTIN

Figure 4H

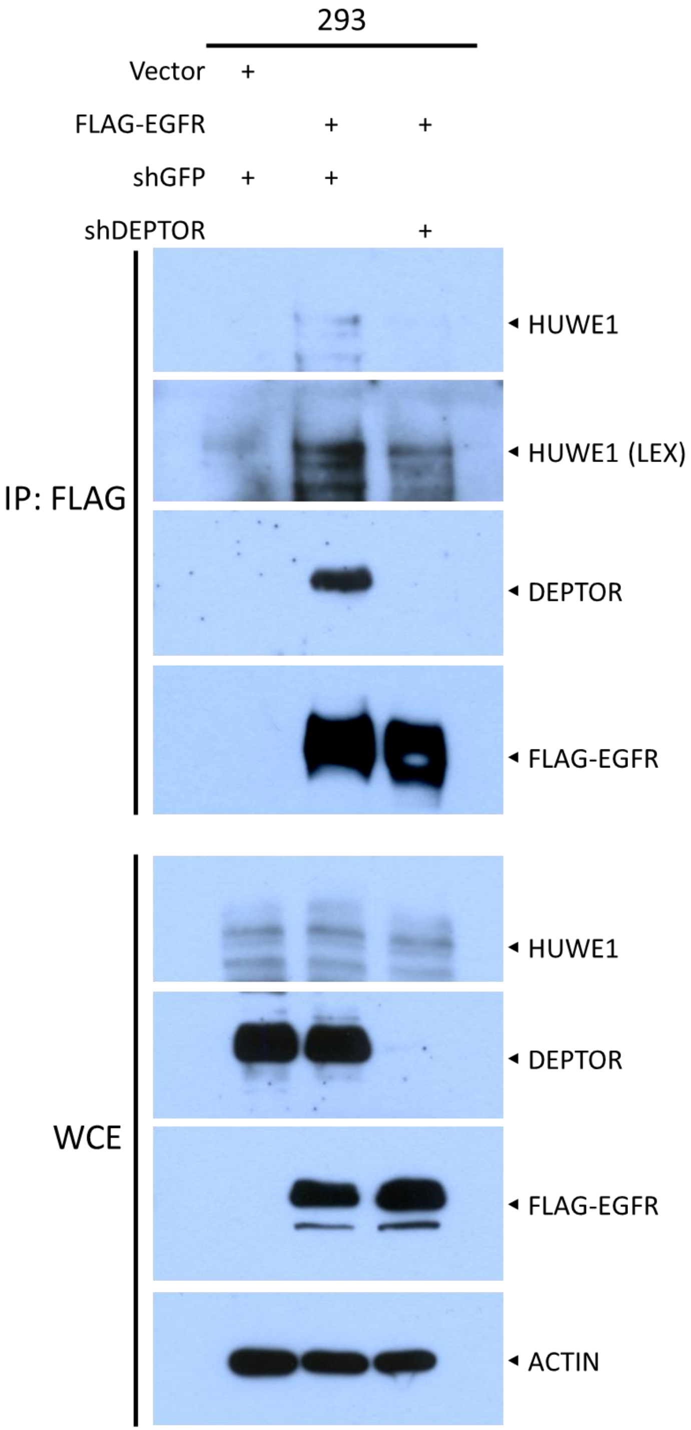
293
Vector +
FLAG-EGFR + +
shGFP + +
shDEPTOR +
IP: FLAG
HUWE1
HUWE1 (LEX)
DEPTOR
FLAG-EGFR
WCE
HUWE1
DEPTOR
FLAG-EGFR
ACTIN

Figure 4I

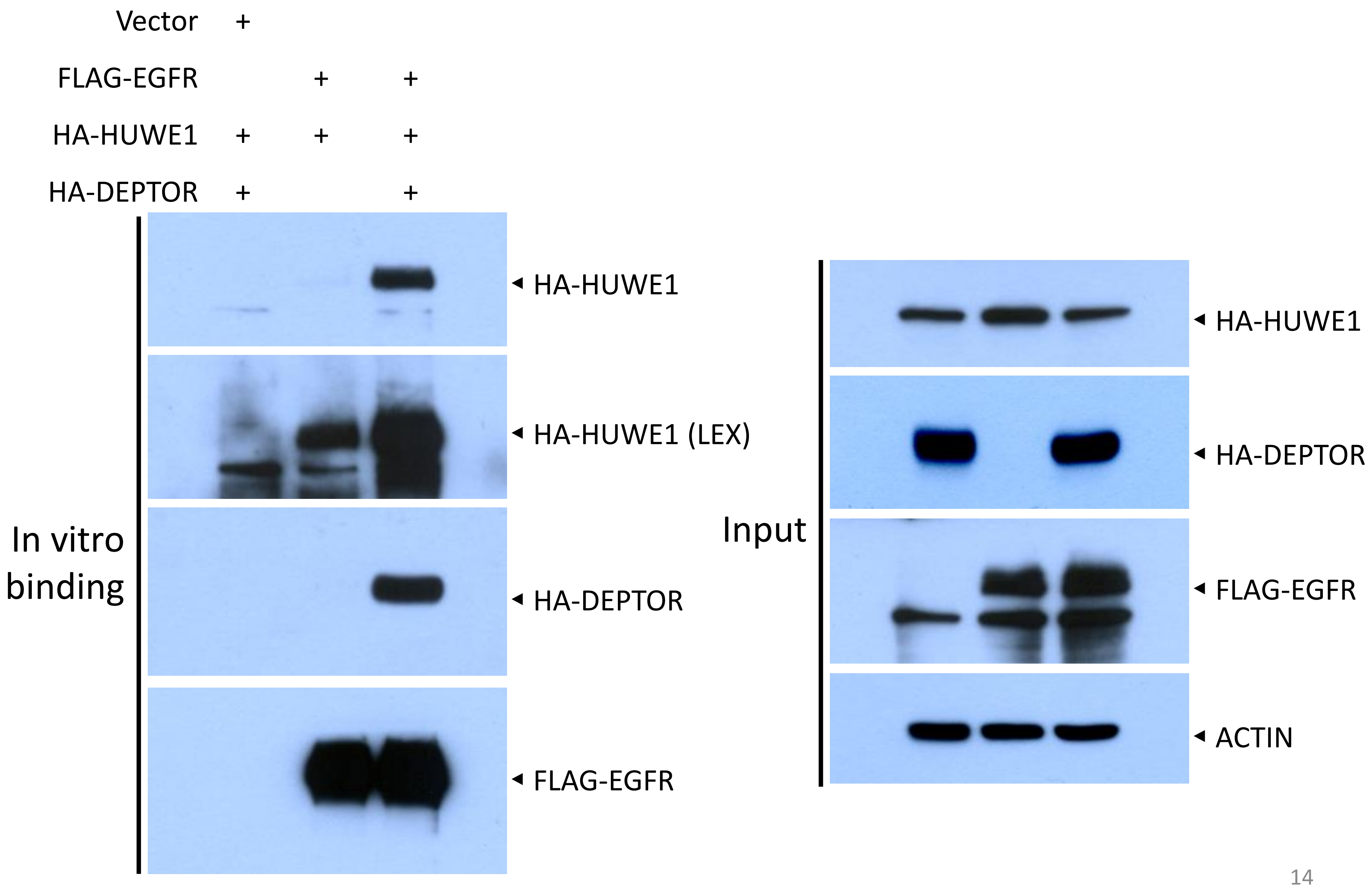
Vector +
FLAG-EGFR + +
HA-HUWE1 + + +
HA-DEPTOR + +
In vitro binding
HA-HUWE1
HA-HUWE1 (LEX)
HA-DEPTOR
FLAG-EGFR
Input
HA-HUWE1
HA-DEPTOR
FLAG-EGFR
ACTIN

Figure 7B

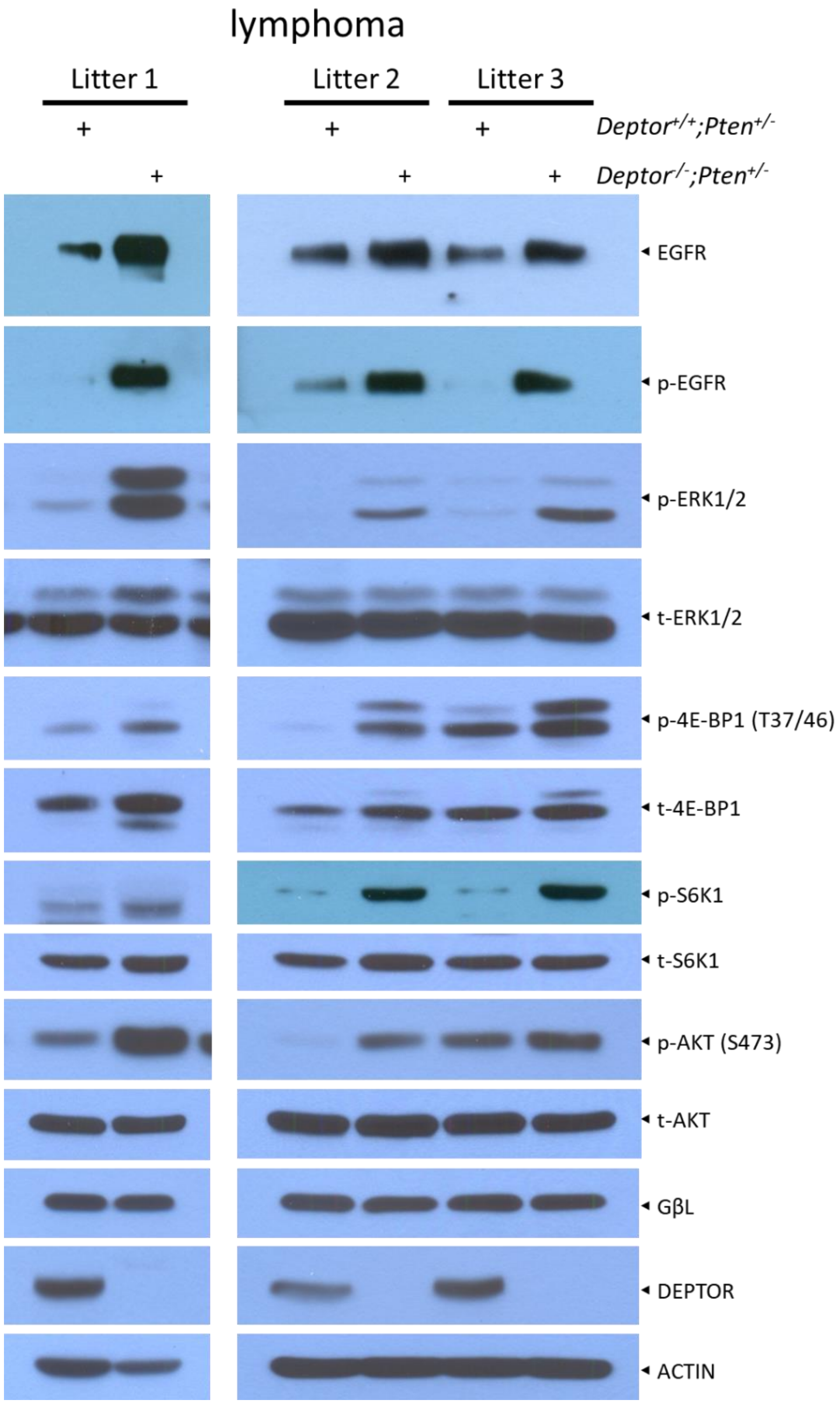
lymphoma
Litter 1
Litter 2
Litter 3
+ + + Deptor+/+;Pten+/-
+ + + Deptor-/-;Pten+/-
EGFR
p-EGFR
p-ERK1/2
t-ERK1/2
p-4E-BP1 (T37/46)
t-4E-BP1
p-S6K1
t-S6K1
p-AKT (S473)
t-AKT
GβL
DEPTOR
ACTIN

Figure S1D

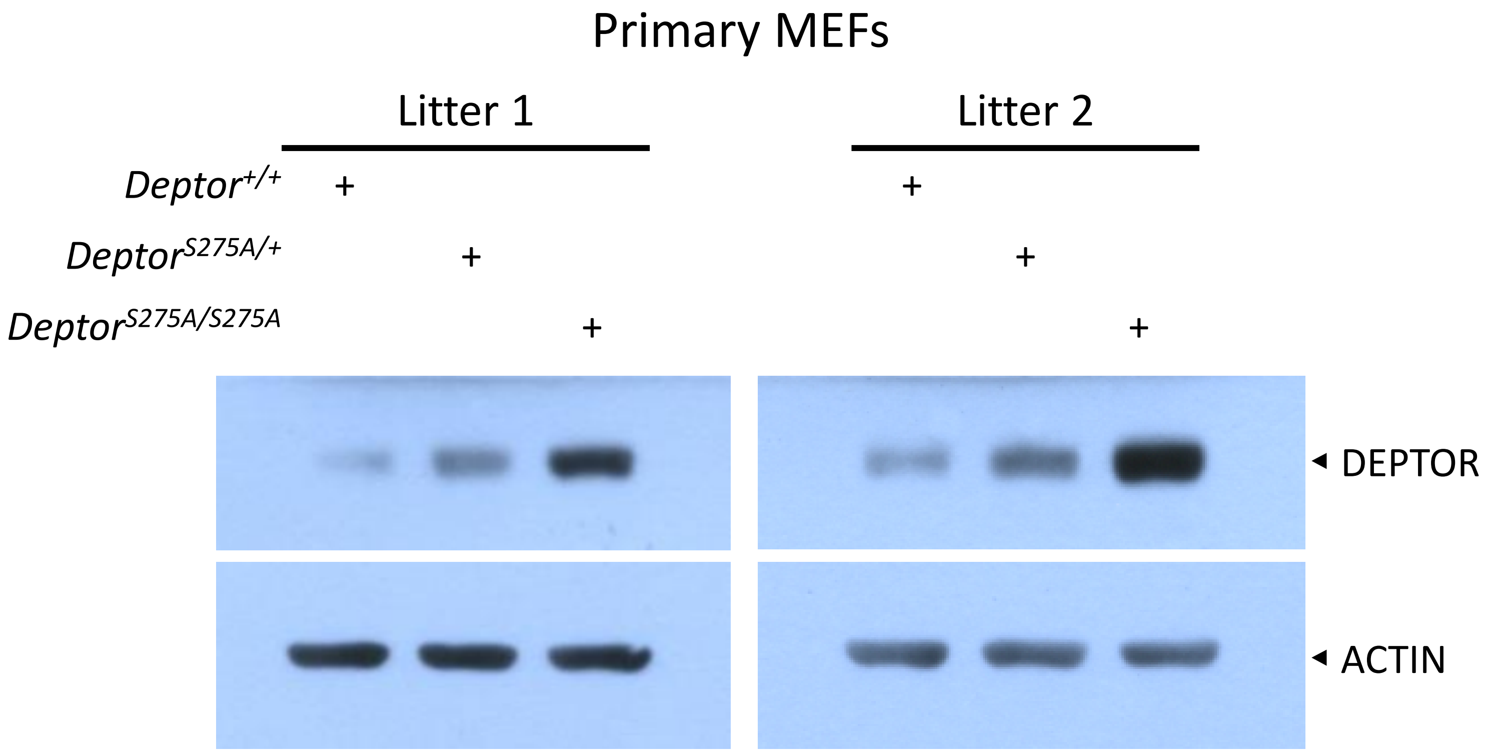

Primary MEFs
Litter 1
Litter 2
Deptor+/+
DeptorS275A/+
DeptorS275A/S275A
+
+
+
+
+
+
DEPTOR
ACTIN

Figure S1E

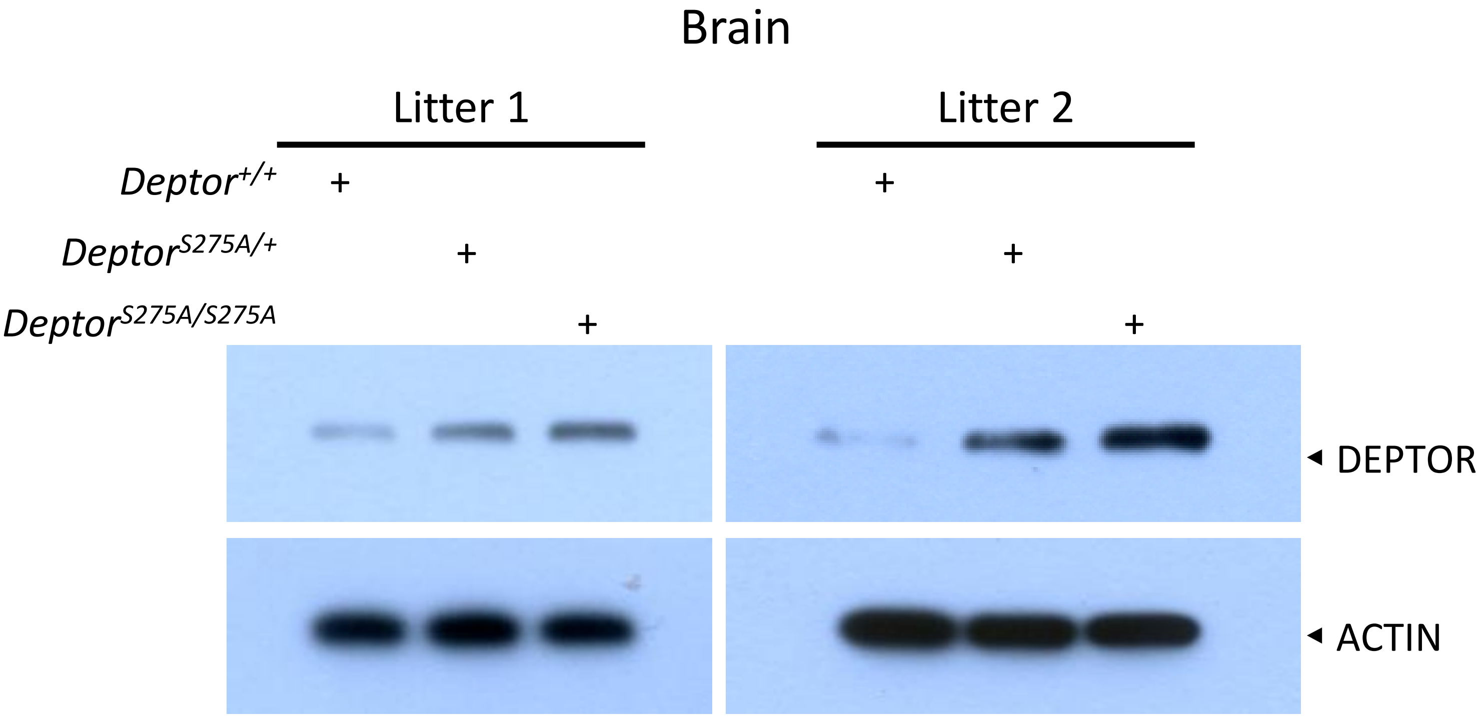
Brain
Litter 1
Litter 2
Deptor+/+
+
+
DeptorS275A/+
+
+
DeptorS275A/S275A
+
+
DEPTOR
ACTIN

Figure S1F

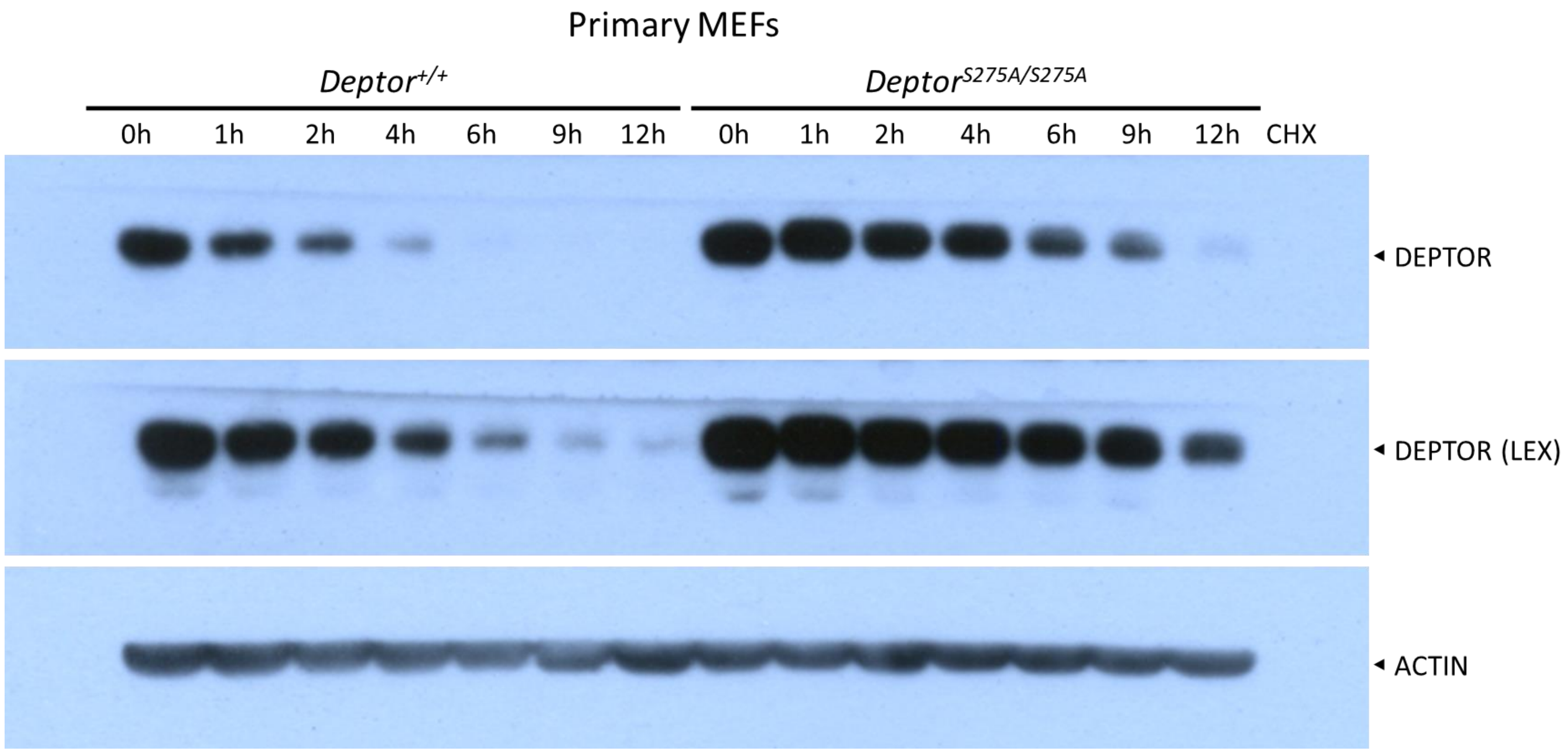
Primary MEFs
$Deptor^{+/+}$
$Deptor^{S275A/S275A}$
0h 1h 2h 4h 6h 9h 12h 0h 1h 2h 4h 6h 9h 12h CHX
DEPTOR
DEPTOR (LEX)
ACTIN

Figure S3A

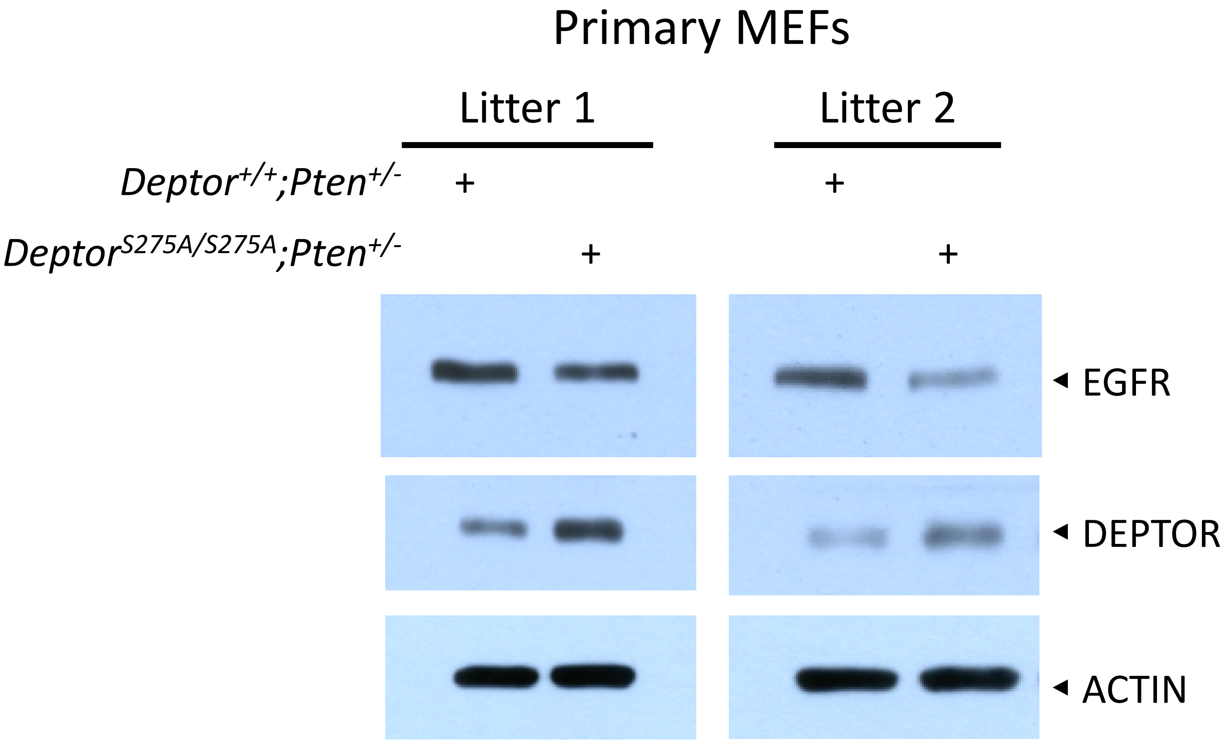
Primary MEFs
Litter 1
Litter 2
Deptor+/+;Pten+/-
+
+
DeptorS275A/S275A;Pten+/-
+
+
EGFR
DEPTOR
ACTIN

Figure S3B

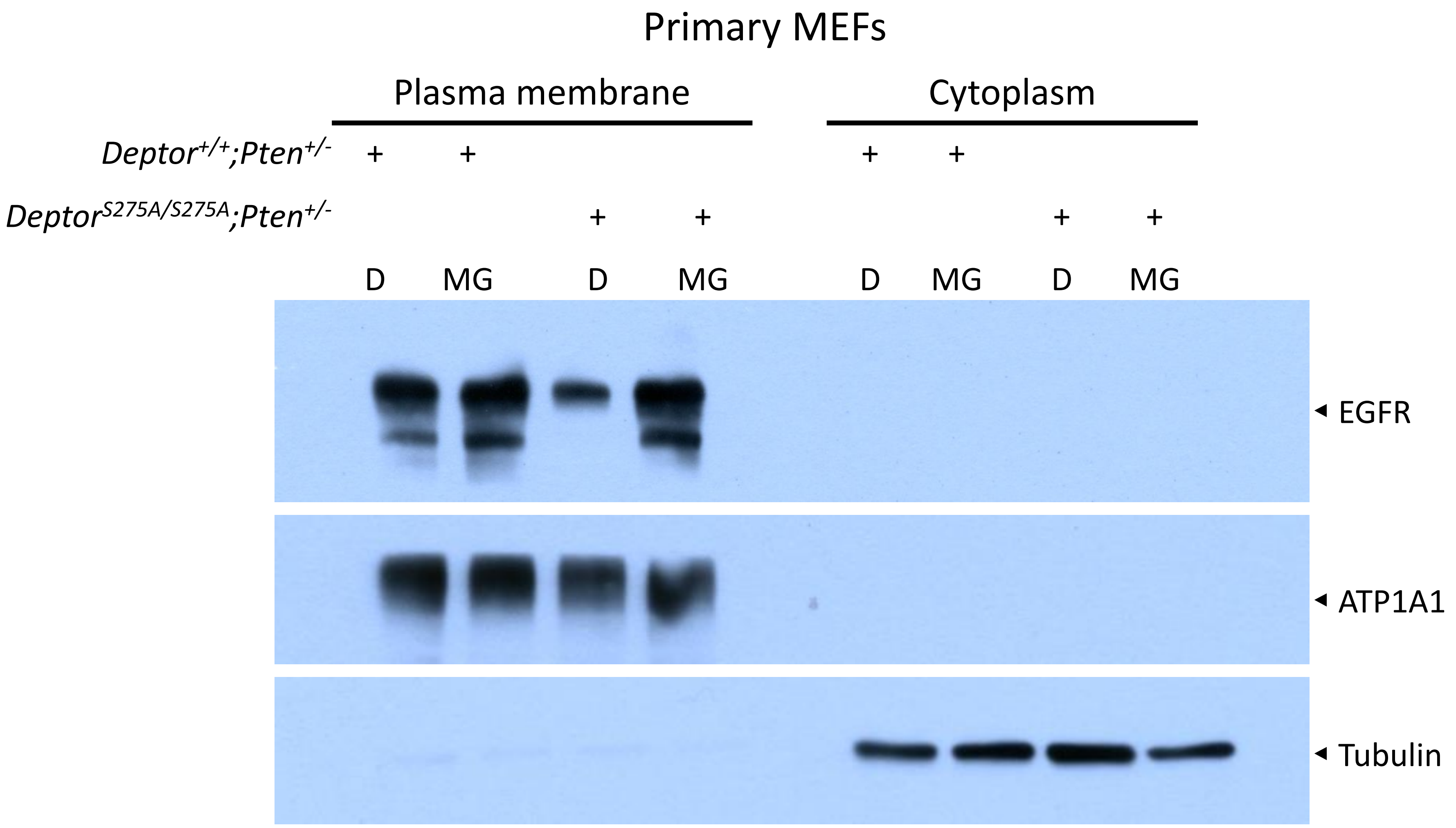

Primary MEFs
Plasma membrane
Cytoplasm
Deptor+/+;Pten+/-
DeptorS275A/S275A;Pten+/-
+
D
MG
EGFR
ATP1A1
Tubulin

Figure S5

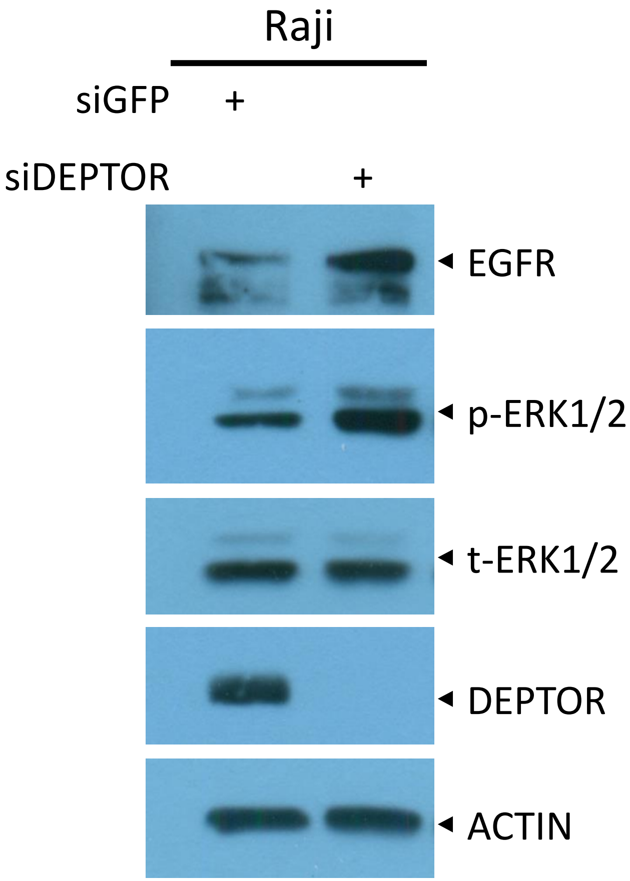

Figure S9A

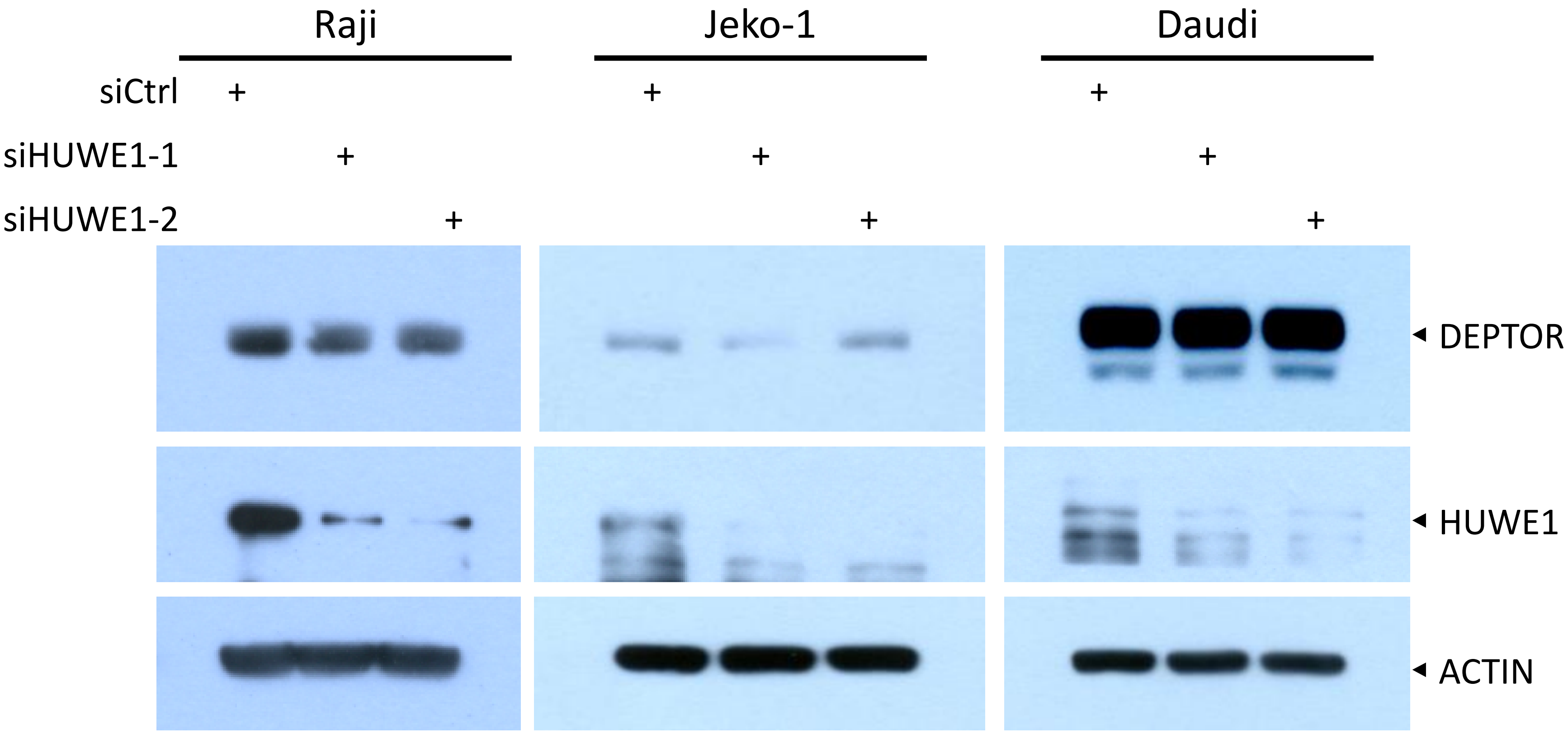

Raji
Jeko-1
Daudi
siCtrl
siHUWE1-1
siHUWE1-2
+
+
+
+
+
+
+
+
+
DEPTOR
HUWE1
ACTIN

Figure S9B

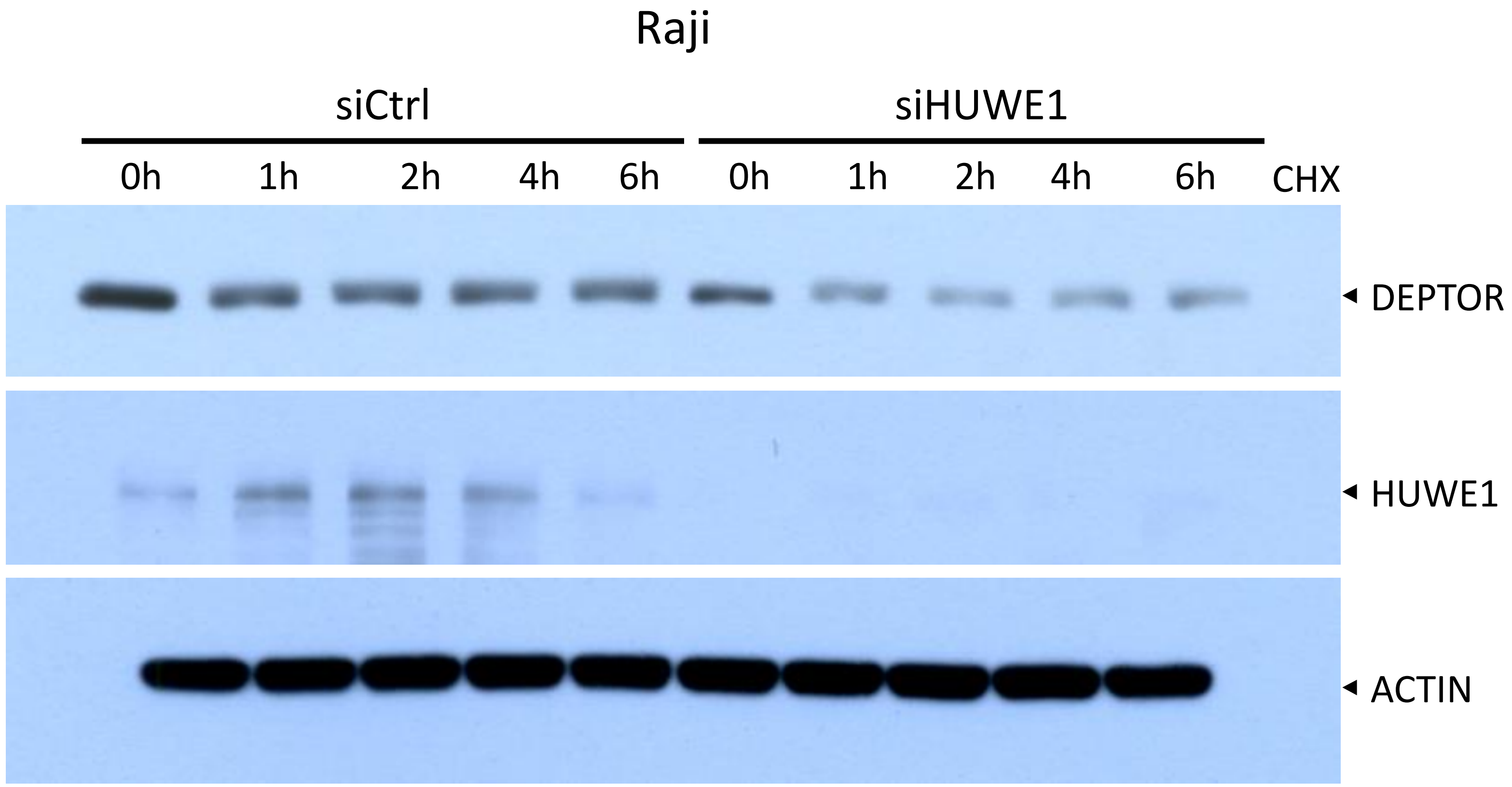

Raji
siCtrl
siHUWE1
0h
1h
2h
4h
6h
0h
1h
2h
4h
6h
CHX
DEPTOR
HUWE1
ACTIN
